# Large Scale RNAi Reveals the Requirement of Nuclear Envelope Breakdown for Nuclear Import of Human Papillomaviruses

**DOI:** 10.1371/journal.ppat.1004162

**Published:** 2014-05-29

**Authors:** Inci Aydin, Susanne Weber, Berend Snijder, Pilar Samperio Ventayol, Andreas Kühbacher, Miriam Becker, Patricia M. Day, John T. Schiller, Michael Kann, Lucas Pelkmans, Ari Helenius, Mario Schelhaas

**Affiliations:** 1 Emmy-Noether Group: Virus Endocytosis, Institutes of Molecular Virology and Medical Biochemistry, ZMBE, University of Münster, Münster, Germany; 2 Cluster of Excellence EXC1003, Cells in Motion, Münster, Germany; 3 Institute of Molecular Life Sciences, University of Zurich, Zurich, Switzerland; 4 Institute of Biochemistry, ETH Zurich, Zurich, Switzerland; 5 Laboratory of Cellular Oncology, National Cancer Institute, National Institutes of Health, Bethesda, Maryland, United States of America; 6 Laboratoire de Microbiologie Fondamentale et Pathogénicité, Université Bordeaux Segalen, Bordeaux, France; Louisiana State University Health Sciences Center Shreveport, United States of America

## Abstract

A two-step, high-throughput RNAi silencing screen was used to identify host cell factors required during human papillomavirus type 16 (HPV16) infection. Analysis of validated hits implicated a cluster of mitotic genes and revealed a previously undetermined mechanism for import of the viral DNA (vDNA) into the nucleus. In interphase cells, viruses were endocytosed, routed to the perinuclear area, and uncoated, but the vDNA failed to be imported into the nucleus. Upon nuclear envelope perforation in interphase cells HPV16 infection occured. During mitosis, the vDNA and L2 associated with host cell chromatin on the metaphase plate. Hence, we propose that HPV16 requires nuclear envelope breakdown during mitosis for access of the vDNA to the nucleoplasm. The results accentuate the value of genes found by RNAi screens for investigation of viral infections. The list of cell functions required during HPV16 infection will, moreover, provide a resource for future virus-host cell interaction studies.

## Author Summary

Certain human papillomaviruses (HPV) are the etiological cause of cervical cancers and other epithelial tumors. Recent advances in the development of anti-HPV vaccines and their increasing deployment provide hope for a significant decrease of these cancers in the future. However, many details of the transmission of HPV between infected and uninfected individuals are still not well understood. In particular, the multistep process of entry into host cells is only partially characterized. Here, we used a systematic RNAi silencing approach to identify host cell proteins required during HPV16 infection, the most prevalent cancer-causing HPV. Our results indicated that HPV entry requires cells to divide. Cell division is important, since it removes the barrier between the nucleoplasm and the cytosol, so that the virus can access the nucleus for transcription and replication. Our study provides new mechanistic insights into the entry process of an important pathogen, and explains why it can only infect the stem cells or transiently amplifying cells of human skin or mucosa. This work also highlights a novel nuclear import strategy for DNA viruses, which typically use the nuclear import machinery to access the nucleoplasm for infection, Thus, HPV entry displays another unique aspect uncommon to most other viruses.

## Introduction

Papillomaviruses are small, nonenveloped DNA viruses that cause mostly benign lesions [1]. Several human papillomavirus (HPV) types are, however, etiological agents for anogenital cancers, and tumors of the head and neck [1]. Among them, HPV16 is the most prevalent, and the best studied [2]. No specific drug treatment against HPV-related cancers exists, but the increasing deployment of anti-HPV vaccines will likely help to reduce the incidence rate of the vaccine types [3]. The spherical HPV particles have an icosahedral (T = 7) architecture, and a diameter of 50–55 nm. The capsid is formed by 72 homo-pentamers of the major structural protein, L1, and contains in addition 12–72 copies of the minor structural protein, L2 [4]. The 8kB DNA genome is circular and double-stranded. During initial infection, HPVs enter basal stem cells or transiently amplifying cells in squamous epithelia [1]. Replication and assembly of new viruses occur in the nucleus, when the host cells differentiate into spinous and granular keratinocytes [1].

Since authentic viruses are difficult to be propagated in sufficient amount and purity, most of what is known about early interactions between HPV and host cells has been learned using virus-like particles (VLPs) and pseudoviruses (PsV). These surrogate particles have the protein composition and architecture of the authentic HPV, but do not carry the viral genome[5], [6]. Instead, the PsV contain a pseudogenome that, when delivered to cells, expresses a reporter protein [6], [7].

Binding of VLPs and PsV of HPVs to tissue culture cells depends on heparan sulfate proteoglycans (HSPG) [8]–[11]. Highly sulfated domains of the HS induce the first of several conformational changes in the incoming virion [12]–[14]. The next change is caused by cyclophilin B, a peptidylprolyl isomerase [15], [16]: The N-terminal peptide of L2 buried within the capsid is exposed so that it can be cleaved by furin [14], [15], [17], [18]. The alterations in the capsid lead to a loss of affinity to HSPGs and transfer to a secondary receptor [8], [14], [15], [19], [20], such as alpha6-integrin, annexin A2 heterotetramer, growth factor receptors, and the tetraspanin CD151 [21]–[28]. However, the exact functions of these receptors in PV entry is disputed [29].

HPV entry into host cells requires endocytosis. Compared with other animal viruses, endocytic uptake is slow and asynchronous (t_1/2_  =  4–12 h)[8], [30], [31]. Reporter gene expression of the pseudogenome is not detected until 24–48 h postinfection (p.i.) [31]. The endocytic mechanism used by high-risk HPV16, 18, and 31 is ligand-induced, clathrin-, caveolin-, lipid raft-, and dynamin-independent, and critically dependent on actin dynamics [27], [31], [32]. The viruses are internalized in small vesicles, transported to late endosomes or endolysosomes, and the Golgi complex [31], [33], [34].

The subsequent steps are poorly understood. It is known, however, that a late step involving penetration into the cytosol or initial uncoating of the capsid requires exposure of the virus to low pH [31]. Also, the major capsid protein L1 dissociates from a subviral complex composed of the viral DNA (vDNA) and L2 probably within endosomal compartments [33], [35]. In transit to the nucleus, the subviral complex is routed to the Golgi complex [33], [34]. After penetration into the cytosol [36], the vDNA moves complexed with L2 into the nucleus and eventually localizes to ND10 nuclear domains [37], [38]. How nuclear import of this subviral complex occurs is not known. Of note, entry of HPV16 requires progression of cells through the cell cycle into early mitosis [39]. However, it is unclear which entry step is influenced by cell cycle progression.

In this study, we used a large-scale RNA interference (RNAi) screen to identify host cell factors and processes involved in HPV entry. Such screens have emerged as a powerful tool to analyze pathogen-host interactions [40]. By focusing our follow-up studies on a cluster of genes that suppresses HPV16 infectivity, we uncovered a crucial role for mitosis in HPV16 nuclear entry. Mitosis was important, since nuclear import of the HPV16 vDNA genome requires changes in nuclear envelope (NE) permeability facilitated by nuclear envelope breakdown (NEB). These changes allowed association of the subviral complex with host cell chromatin aligned on the metaphase plate.

## Results

### RNAi screening

**Figure 1:**
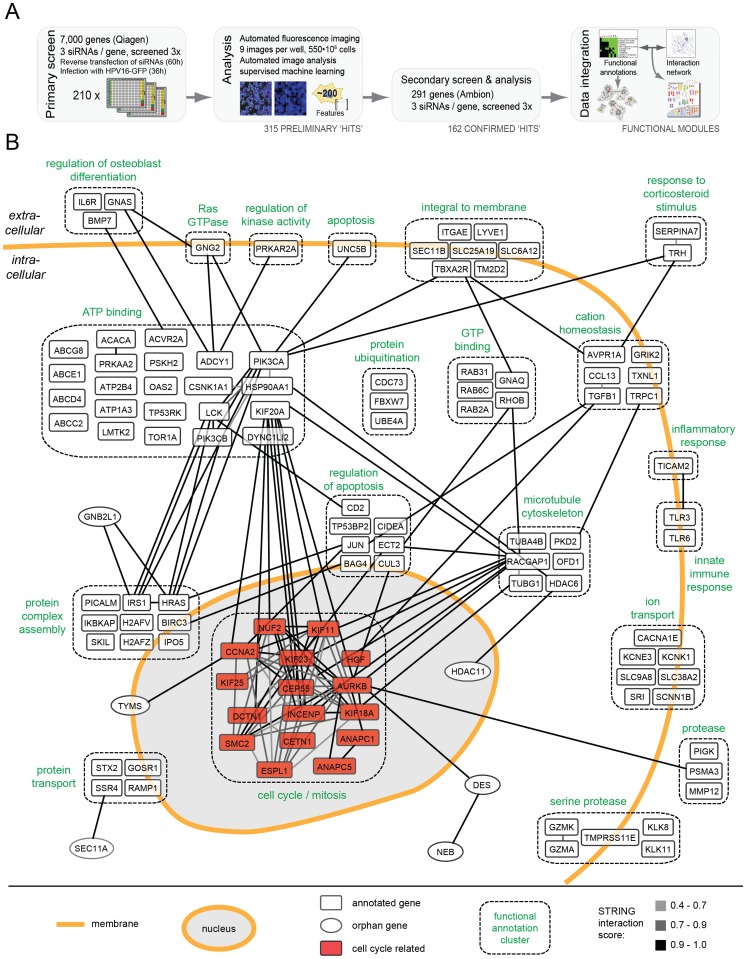
HPV16 RNAi screen and map of host cell factors involved in entry. (A)Outline of the RNAi screening and bioinformatic procedures. (B) 159 hits (by gene symbol) that enhanced or decreased HPV16 infection after silencing were assigned to enriched functional annotation clusters. Functional clusters with simplified annotations are depicted as boxes with dashed outlines (compare Tables S1 and S2). Connecting lines between genes reflect the degree of confidence of interaction (high  =  black, i.e. 0.9–1.0; intermediate  =  dark gray, i.e. 0.7–0.9, low  =  light gray, i.e. 0.4–0.7). The mitosis cluster is highlighted in red.

To identify cellular factors and processes involved in HPV16 entry, we performed an automated, high-content, high-throughput RNAi screen in human epithelial cells (HeLa) (Figure 1A). For infection, PsV were used that contained a pseudogenome encoding the green fluorescent protein (GFP) under the control of the cytomegalovirus (CMV) early promoter (HPV16-GFP). Expression of GFP allowed identification and quantification of cells in which the pseudogenome had been successfully delivered to the cytosol, uncoated, imported into the nucleus, transcribed, and translated.

The number of cells and the fraction of GFP-expressing cells were determined 36 h p.i. using automated microscopy (Figure S1). The multiplicity of HPV16 was kept low so that only about 10% of the cells in control wells were positive. This allowed us to not only identify genes required for infection, but also those that act as enhancer genes, i.e. proteins that increased the fraction of infected cells when depleted. Moreover, using a low amount of siRNA (5 nM), we aimed to minimize off-target effects and reduce the number of false-positive hits. To increase reliability, we further used a fully automated computational pipeline for high-content image analysis [41]. Several iterations of supervised machine learning were applied to identify virus infection, mitosis, apoptosis, and technical phenotypes.

The RNAi screen was carried out in two steps. Three non-overlapping siRNAs against each gene were used in a primary screen, and three additional siRNAs in a secondary follow-up screen. In the primary screen, 7,000 human genes selected for the potential to be inhibited by small compounds (the druggable genome library, Qiagen) were screened. The library encompasses a well-studied and well-annotated cross-section of genes. Most of the genes have known functions, and many inhibitors and other reagents are readily available for follow-up studies. The primary screen, which was performed three times, led to the identification of 315 genes in which the median of the three siRNAs reproducibly reduced or enhanced infection by at least 3 robust z-scores (Table S1, all). To provide a resource for future studies of HPV-host cell interaction, the full set of images and analysis results is available at http://www.infectome.org.

The follow-up screen was directed against the genes identified as hits in the primary screen. It made use of three additional siRNAs per gene from another vendor (Ambion) except for a few genes against which no additional siRNAs were available (Table S1, red). The secondary screen was also repeated three times. It confirmed 162 (56%) of the tested 291 hits from the primary screen, when the same criteria as the primary screen were applied.

The final hit list represented a broad range of cell functions (Table S1). Among these functions, several have been previously implicated in HPV16 infection: e.g. phosphoinositide 3-kinase (PI3K) signaling (represented by PI3K subunits PIK3CA, PIK3CB), sodium/proton exchange (SLC9A6, SLC9A8, SCNN1B), minus-end directed microtubular transport (motor complex dynein (DYNC1H1, DYNC1L1I2); microtubule-regulating kinases (MARK1, MASTL)), tyrosine kinase signaling (LMTK2, LCK), dephosphorylation (phosphatases (INPP5B, PtPN4, PPP2R5C, ACP5)), signaling by the small G-protein Ras (HRAS, RASA4), and the matrix metallopeptidases (MMP12) [21], [31], [42]–[44]. Other hits, such as metabolic and catabolic proteins, or of the biosynthetic machinery were expected for any virus. However, the majority of hits had no prior connection to HPV16 infection.

To extract information regarding critical processes in HPV entry, the list was subjected to rigorous bioinformatic analysis. An algorithm that combined function- and interaction-based information was used [45]. When assigned to functional annotation clusters representing sets of proteins that share common annotations within public databases [DAVID; [46]], the hits were enriched within 32 clusters and several highly connected, functional annotation networks (Table S2). The function-based information was combined next with data on protein interactions among individual host factors [STRING; [47]].

The result of the combined analysis is shown for 117 host factors in Figure 1B (compare Figure S2, in which the hits were subdivided by their effect, i.e. reducing or enhancing infection upon knockdown). Automatic annotation was used to visualize the interaction networks (gray to black lines) both within and between functional annotation clusters (dashed boxes). A high number of cellular regulators appeared to be hits, ranging from receptor molecules, kinases, transporters, and ion channels. Since the regulation of the cellular processes leading to entry, such as endocytosis and intracellular trafficking are poorly characterized with regard to signal transduction and the influence of ion flux regulation, these findings are likely to open new avenues in HPV and cell biological research.

To us the most interesting was a cluster of mitosis regulators such as AURKB, ANAPC and INCENP. It showed many interconnections within and outside the cluster (Figure 1B). The high abundance of genes and multiple interconnections among them highlighted the importance of this cluster. Interestingly, most of these genes enhanced infection upon knockdown (Figure S2A). In contrast, genes in a cluster known to regulate nucleoplasm architecture such as HDAC11, CCNE2, and POLI reduced infection upon depletion (Table S2).

### Infection is completed during mitosis

**Figure 2:**
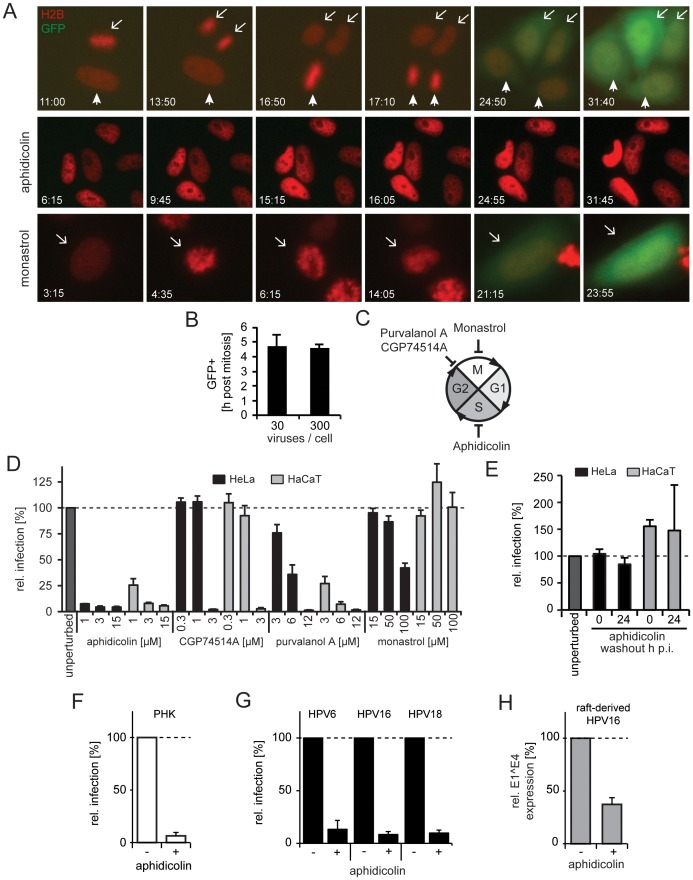
Cell cycle progression into early mitosis is required for HPV16 entry. (A) Depicted are images from representative time lapses for untreated (first row), aphidicolin-treated (second row), and monastrol-treated (last row) HeLa H2B-mCherry cells infected with HPV16-GFP (30vge/cell, see also Videos S1–S3). Time stamps (hh:mm) indicate the time after virus addition. Mitoses in untreated cells are marked by arrows/arrowheads. Note that monastrol-treated cells failed to separate chromosomes but underwent chromosome condensation, aberrant metaphase plate formation, and decondensation of chromosomes (arrows). (B) as in (A). To relate the timing of mitosis and the onset of GFP expression (i.e. successful HPV16 entry), HeLa H2B-mCherry cells were infected with HPV16 (30 and 300 vge/cell), and cells were imaged by time-lapse microscopy. The end of mitosis was defined by visible chromosome decondensation (i.e. telophase), whereas the onset of GFP expression was defined as 10% signal above background. Given is the relative timing of GFP onset post mitosis ± standard deviation (SD). (C) Schematic depiction of the cell cycle. The effects of the inhibitors on cell cycle transitions are indicated. (D) HeLa (black bars) or HaCaT (gray bars) cells were infected with HPV16 PsV after 16 h of preincubation in the presence of inhibitors or solvent control (unperturbed) at indicated concentrations. The number of infected cells was determined 48 h p.i. by flow cytometry. Depicted are infected cells relative to control in percent ± SD. (E) as in (D). To test the reversibility of the S-phase arrest for infection, cells were treated with aphidicolin, and the drug was washed out at the time of HPV16 infection (0 h p.i.) or 24 h p.i.. (F) as in (D) using PHK cells. (G) HeLa cells were infected with HPV6, HPV16, or HPV18 PsV after 16 h preincubation aphidicolin. The number of infected cells was determined 48 h p.i. by flow cytometry. Depicted are infected cells relative to the DMSO treated control in percent ± SD. (H) HaCat cells were infected with raft-derived HPV16 after 16 h preincubation with aphidicolin. Total RNA was extracted 48 h p.i., and infection was measured by the viral splice transcript E1∧E4 normalized to TBP. Infection is depicted relative to the DMSO treated control in percent ± SD.

To follow up on the involvement of the mitotic and cell cycle regulators in HPV16 entry, we first characterized the temporal relationship between mitosis and HPV infection. For this, we followed the GFP expression from HPV16-GFP in relation to chromosome condensation, segregation, and decondensation in HeLa cells that stably express the histone 2B tagged with mCherry (H2B-mCherry [48]). It was clear from this analysis that GFP expression only occurred if mitosis had taken place as monitored by chromosome segregation (Figure 2A first row, Video S1). In fact, the GFP signal was first detected on average 4.5 h after chromosome decondensation in the newly formed nuclei (Figure 2B). Given that expression and functional chromophore formation of the GFP *in vitro* require 0.5 and 3–3.5 h, respectively [49], the time course suggested that the establishment of HPV infection correlated with completion of mitosis. That mitosis defined the onset of viral gene expression was further supported by the observation that the onset of GFP expression did not change with the number of pseudogenomes delivered into cells (Figure 2B).

### Entry into early mitosis is required for infection

Next, we aimed to relate our finding that mitosis defined the onset of viral gene expression to previous findings that indicated the requirement for cell cycle progression into early mitosis for infection [39]. To corroborate the cell cycle requirements for infection in HeLa and HaCaT cells, we used inhibitors known to block at three specific stages. Progression in the synthesis (S-)phase was blocked by aphidicolin, gap2-phase to mitosis (G2/M) transition by purvalanol A or CGP74514A, and chromosome segregation/cytokinesis during mitosis by monastrol (Figure 2C). As expected, the inhibitors caused significant changes in the fraction of cells in G1-, S-, and G2/M-phase (Figure S3).

Arresting cells in S-phase by aphidicolin blocked infection efficiently (Figure 2D) and reversibly (Figure 2E). In line with our observations in fixed cells, GFP expression could not be detected in live cells arrested in S-phase (Figure 2A middle row, Video S2). This effect was not limited to transformed cells, as infection of primary human keratinocytes (PHK) was blocked by aphidicolin (Figure 2F). S-phase arrest by aphidicolin also blocked infection of HPV6 and HPV18 PsV, and of raft-derived HPV16 (Figure 2G, H). Together, this suggested that cell cycle progression through S-phase was required for infection of various HPV types independent of whether PsV or raft-derived virions were used for infection of transformed or primary cells. Similar to S-phase arrest, inhibition of the G2/M transition by purvalanol A and CPG74514A prevented infection (Figure 2D). In contrast, if mitosis was interrupted at the stage of chromosome segregation by monastrol treatment, GFP expression did occur (Figure 2D). Similarly, GFP expression occurred in live cells treated with monastrol (Figure 2A last row, Video S3). Monastrol prevents monopolar spindle formation after NEB by inhibiting the kinesin KIF11 [50], [51]. This was also obvious in the live HeLa cells, which featured condensation, erratic alignment, and decondensation of chromosomes consistent with mitotic progression into prometaphase/metaphase followed by a block in chromosome segregation and cytokinesis. Thus, our results corroborated that early steps in mitosis were important for HPV16 entry, but later steps such as spindle segregation and cytokinesis were not. The early stages of mitosis are complex and highly regulated. They involve changes in cytoskeletal organization, vesicular transport, permeability of the nuclear envelope induced by NEB, and transcriptional regulation [52]. As hypothesized previously [39], any of these changes may affect HPV16 entry.

### Nuclear import of HPV16 is blocked in interphase cells

Earlier studies indicated that HPV16 particles bind to all cells in a population [19], [31], [53], i.e. irrespective of their cell cycle phase. The key question therefore was which step in the entry program of the virus is inhibited in interphase cells. To study this question, we followed the fate of the PsV in aphidicolin-treated, S-phase-arrested cells and analyzed the efficacy of virus endocytosis, intracellular trafficking of viruses to the perinuclear area, viral uncoating, and nuclear import of vDNA.

**Figure 3:**
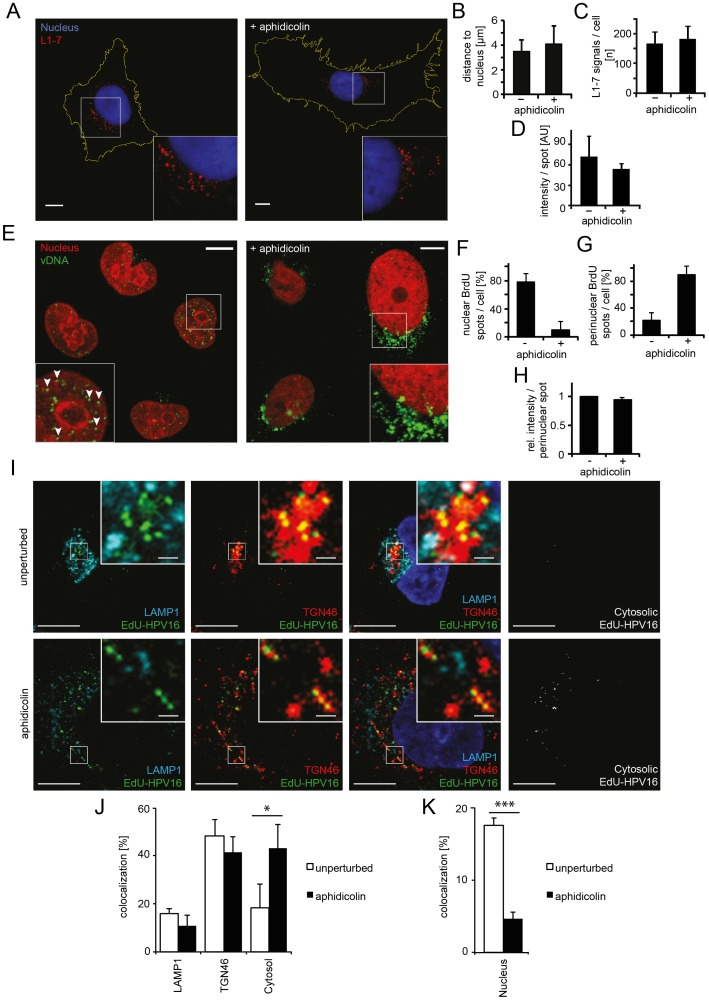
Nuclear import of HPV16 vDNA is blocked in interphase cells. (A) Aphidicolin-treated or untreated HeLa cells were infected with HPV16-GFP. At 16 h p.i., cells were fixed and immunostained with the L1-7 antibody detecting endosomal, conformationally altered virions. Depicted are confocal sections of untreated (left) or aphidicolin-treated (right) cells. (B) As in (A) with quantification of the three-dimensional distance of L1-7 signals to the nuclear border (in µm) ± SD. (C) As in (A) with quantification of the number of discernible L1-7 spots/cell. (D) As in (A) with quantification of the fluorescent intensity (in arbitrary units, AU) of individual L1-7 spots. (E) HeLa H2B-mCherry cells were infected with BrdU-HPV16. Cells were immunostained for BrdU to detect the vDNA after fixation at 24 h p.i.. Depicted are confocal sections of aphidicolin-treated (interphase, right) or untreated (left) cells. Mostly, the vDNA localized intranuclearly as discrete spots in untreated cells (arrowheads) or exclusively perinuclearly in aphidicolin-treated cells. (F) As in (E) with quantification of intranuclear BrdU spots/cell. The number of intranuclear spots is given relative to the total number of cellular spots. (G) As in (E) with quantification of perinuclear BrdU spot/cell as in (F). (H) As in (E) with quantification of the signal intensities of individual perinuclear BrdU spots relative to untreated infected cells. (I) HeLa cells were treated with or without aphidicolin for 16 h prior to infection. 20 h after infection with EdU-HPV16 (green), cells were fixed and immunostained for TGN46 (red) and LAMP1 (light blue). The nucleus was stained by Hoechst (dark blue). Depicted are single confocal sections. The cytosolic EdU-HPV16 signal is depicted after substraction of the EdU-HPV16 signals colocalizing with LAMP1, TGN46 and the nucleus. (J) As (I) with quantification of signal colocalization of EdU-HPV16 with LAMP1 or TGN46. Cytosolic EdU-HPV16 amounts were defined as signals that did not colocalize with LAMP1, TGN46, or the nucleus (see below). (K) As in (I) with quantification of signal colocalization of EdU-HPV16 with the nuclear stain (Hoechst). Statistical significance was determined by a two-tailed, independent t-test; P-values: * <0.05; *** <0.001. All scale bars: 10 µm.

First, the endocytic uptake of fluorescently labeled particles into cells was analyzed in S-phase arrested cells by flow cytometry. Internalization was unaffected (Figure S4A). When a monoclonal antibody against L1 (L1-7) that detects partially uncoated particles but not mature virions [27], [54] was used to localize the virus, a perinuclear accumulation was observed in both control and S-phase-arrested cells (Figure 3A). This indicated that intracellular trafficking and partial uncoating of the endocytosed viruses occurred as efficiently as in unperturbed cells. Neither the number and the intensity of fluorescent spots, nor their 3D distance from the nuclear border significantly differed in cells arrested in S-phase (Figure 3B–D). An inhibitor exchange experiment indicated moreover that the incoming viruses passed the acid-dependent step in interphase cells as well. In this experiment, infection for 24 h in the presence or absence of aphidicolin was followed by NH_4_Cl treatment for 48 h. Infection in the aphidicolin-treated cells was normal compared to the control (Figure S4B). Since NH_4_Cl prevents low pH-activation of HPV16 in endosomes [31], we concluded that the aphidicolin-induced block occurred after low pH activation of the virus in endosomes or the trans-Golgi-network (TGN).

Finally, the exposure (uncoating) and intracellular localization of vDNA were analyzed. Cells were infected with HPV16 that contained DNA labeled with 5-bromo-2′-deoxy-uridine (BrdU), a nucleoside analog that allows immunodetection after partial uncoating of the DNA [38]. At 24 h p.i., the vDNA was readily detected in the nuclei of infected cells with BrdU-specific antibodies (Figure 3E, left). In cells arrested in S-phase, the vDNA was detectable to a similar extent as in untreated cells (Figure 3E, right, 3H) suggesting that the vDNA was accessible to antibodies. However, the signal was located in the perinuclear area instead of within the nucleoplasm (Figure 3E–G). To more thoroughly analyze the intracellular localization of the viral DNA in aphidicolin-treated cells, PsV containing 5-ethynyl-2′-deoxyuridine (EdU)-labeled vDNA (EdU-HPV16) were used for infection, which allowed a more sensitive detection. Irrespective of whether cells were arrested in S-phase or not, the vDNA localized to the same extent to LAMP1-, and TGN46-positive structures (Figure 3I, J). Since LAMP1 and TGN46 are markers for late endosomes and the TGN, respectively, the results supported that vesicular trafficking of the virus was unperturbed. The nuclear localization of EdU-labeled vDNA was clearly reduced in aphidicolin-treated cells (Figure 3K), as observed for the BrdU-labeled vDNA. Instead, the proportion of virus that did not localize to LAMP1- and TGN46-positive structures, or the nucleus, increased significantly (Figure 3J). It is likely that this vDNA signal reflected the localization of the subviral complex to the cytosol after penetration, as our previous results indicated no perturbation in HPV internalization, vesicular trafficking and uncoating.

Our observations, thus, indicated that whereas endocytic uptake of the virus, intracellular transport, acid-activation, and uncoating were normal in interphase cells, the vDNA failed to be imported into the nucleus from the cytosol. Taken together, these data showed that mitosis was required for infection, because it allowed nuclear import of the viral genome.

### HPV16 does not require nuclear pore complexes for nuclear import

Viruses that replicate in the nucleus generally exploit the nuclear import machinery of the cell and nuclear pore complexes in the nuclear envelope [55]–[57]. S-phase arrest typically does not impair nuclear import (and export) through nuclear pore complexes, or of viruses that replicate in the nucleus [58], [59]. However, to rule out a perturbation in nucleo-cytoplasmic shuttling, we infected aphidicolin-treated cells with Herpes Simplex Virus type 1 (HSV-1) that requires functional nuclear pore complexes for import of its DNA genome and subsequent infection [60]. A recombinant HSV-1 that expresses GFP under the control of the same promoter as in the HPV16 PsV reporter constructs was used [7], [61]. It allowed us to monitor effects on gene expression in addition to nuclear import of HSV-1 vDNA.

We found that cells were infected by HSV-1 to the same extent whether the cells were arrested in S-phase or not (Figure S5A, B, Video S4). Therefore, neither the nuclear pore machinery nor the subsequent gene expression appeared to be affected by this treatment. Also, the nuclear import of the importin beta binding domain fused to GFP (IBB-GFP) occurred to a similar extent in unperturbed and S-phase arrested cells (Figure S5C, D) indicating that karyopherin-mediated transport was unaffected.

Since the nucleo-cytoplasmic shuttling appeared not to be impaired under our experimental conditions, it was unlikely that the block in HPV16 vDNA import upon S-phase arrest was caused by perturbation of the nuclear pore complexes. To substantiate this, we impaired nuclear import by depleting NUP153 by RNAi. NUP153 is a nuclear pore protein essential for nuclear pore basket formation, nuclear pore complex anchoring and import of a variety of nuclear proteins [62]. While NUP153 knockdown clearly reduced HSV-1 infection, HPV16 infection was largely unaffected (Figure S5E). Taken together, the data indicated that HPV16 did not require nucleo-cytoplasmic transport via nuclear pore complexes for import of the vDNA.

### Transient loss of NE barrier function facilitates HPV16 infection

So how does the vDNA of HPV16 enter the nucleoplasm? One possibility is that it relies on NEB, which removes the nucleo-cytoplasmic barrier during mitosis. The NE is disassembled during G2/M transition, and reassembles during telophase after segregation of the chromosomes prior to cytokinesis [52]. It is thought that the provirus of certain gamma retroviruses uses this time window to access the nucleoplasm [63].

**Figure 4:**
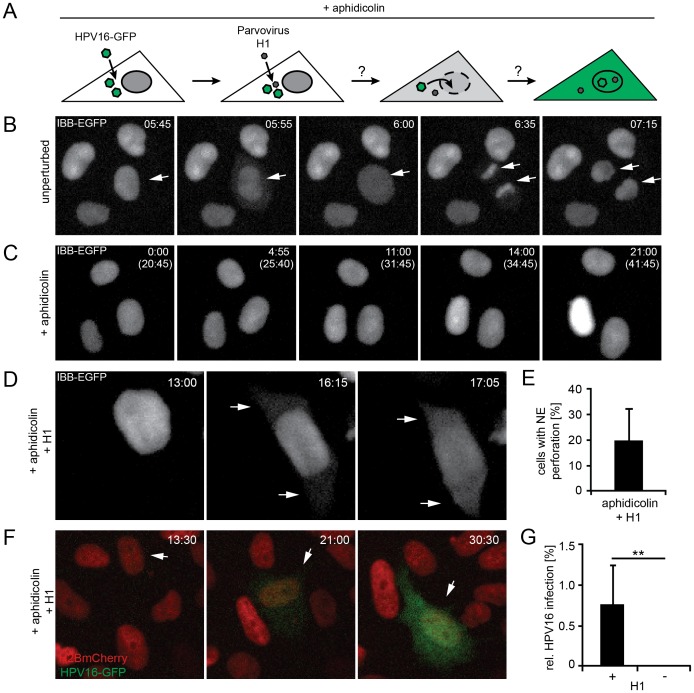
Nuclear envelope perforation allows HPV16 entry in interphase cells. (A) Schematic depiction of the experimental setup. Aphidicolin-treated cells were infected (loaded) with HPV16, after which they were superinfected with parvovirus H1. (B) Time-lapse images of HeLa IBB-GFP cells (see Video S5). Arrowheads indicate mitotic events, during which the IBB-GFP signal dispersed throughout the cytosol after NEB. Indicated is the time after image acquisition was started. (C) As in (B) of cells treated with aphidicolin. Indicated are the times after image acquisition was started and, in brackets, after aphidicolin was added (see Video S6). (D) As in (C) with addition of parvovirus H1. Timestamps indicate the time after H1 addition. Note the translocation of the IBB-GFP signal into the cytosol (arrows, see Video S7). (E) Quantification of video microscopy from (D). Depicted is the relative number of cells exhibiting IBB-GFP translocation to the cytosol (NE perforation by H1) ± SD (18 fields of view/experiment) (F) HeLa H2B-mCherry cells were treated with aphidicolin for 16 h, when cells were infected with HPV16 PsV. 12 h post HPV16 infection, cells were superinfected with parvovirus H1, and time-lapse recording was started. Depicted are images from a representative time lapse (see Video S8). Time stamps indicate the time after H1 addition. (G) Quantification of video microscopy from (F) with or without H1 addition. Depicted are the numbers of infected cells in percent ± SD (n = 350 cells/experiment). Statistical significance was determined by a one-tailed, independent t-test: P-value **<0.002.

If HPV16 uses the same strategy as the gamma retroviruses, one would expect that damage to the integrity of the NE would allow the vDNA resident in the cytoplasm to gain access to the nucleus. To induce perforation of the nuclear membranes without mitosis, we made use of parvovirus H1, a small DNA virus known to enter the nucleus by inducing holes in the NE [64]. These hole are large enough to allow sudden entry of antibodies into the nucleus and, at least in some cells, chromatin leakage into the cytoplasm. To confirm that H1 induced NE perforation in our cells, we monitored the fluorescence signal of IBB-GFP. In unperturbed cells, IBB-GFP was found to be intranuclear during interphase but dispersed in the cytosol during mitosis as a result of NEB, as expected (Figure 4B, Video S5, [65]). The IBB-GFP signal persisted in the nucleus in aphidicolin-treated cells because neither mitosis nor NEB occurred (Figure 4C, Video S6). In contrast when incubated with H1, 20±13% of the aphidicolin-treated cells exhibited translocation of the IBB-GFP signal to the cytosol consistent with the expected extent of NE perforation (Figure 4D, E, Video S7, [64], [65]).

When S-phase-arrested cells were infected with HPV16, and the NE was perforated by superinfection with H1 (Figure 4A), a small but readily detectable number of cells (1±0.5%) was infected suggesting that H1-induced perforation of the NE was sufficient for productive entry of vDNA into the nucleus (Fig 4F, G, Video S8). Without H1 there was no detectable infection (Figure 4G, Video S2). These findings were consistent with the idea that vDNA/L2 complexes cannot enter the nucleus as long as the NE is intact. Therefore, it is likely that mitosis is necessary for infection, because it causes a transient loss of the barrier function provided by the NE. The small number of infected cells after the transient NE perforation indicated that although sufficient, NE perforation is not highly efficient to allow nuclear import. This is likely due to the short, temporary state of NE perforation combined with the restricted diffusion of the subviral complex previously observed for other macromolecular complexes [66].

### The vDNA and L2 associate with host cell chromosomes on the metaphase plate during mitosis

**Figure 5:**
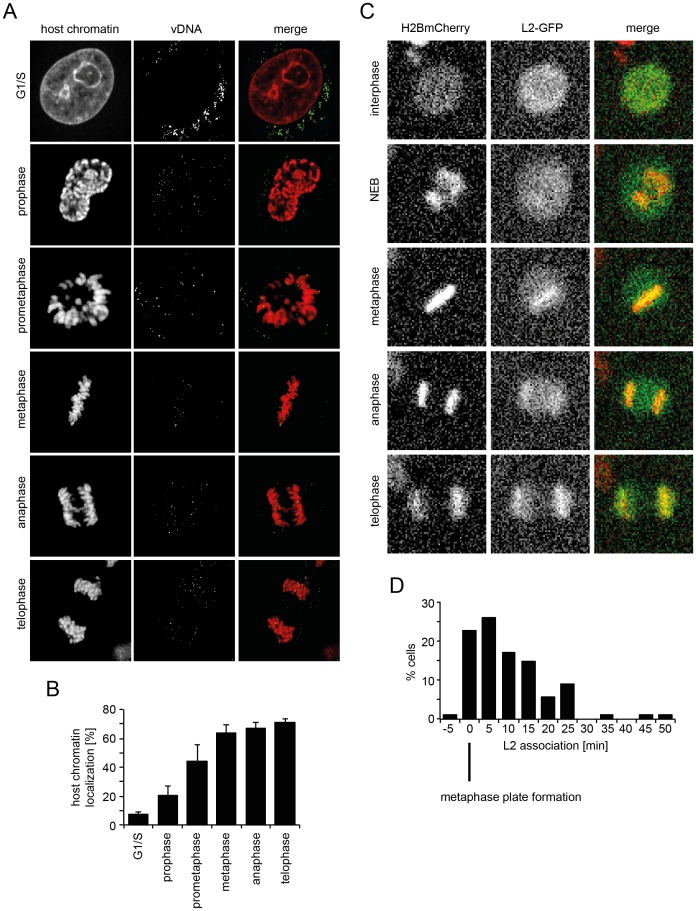
vDNA and L2-GFP associate with mitotic chromosomes. (A) HeLa Kyoto cells were infected with EdU-HPV16. Cells were stained for host DNA and EdU to detect the vDNA after fixation at 20 h p.i.. Depicted are host cell chromatin (left), the vDNA (middle), and merge (right) of confocal sections of an aphidicolin-treated cell (G1/S) and untreated mitotic cells. Note that vDNA commenced association with chromosomes upon entry into mitosis. Indicated are the mitotic stages based on chromosomal organization. (B) as in (A). Quantification of vDNA localization to host chromatin ± SD (n = 5). (C) HeLa H2B-mCherry cells were transiently transfected with an L2-GFP expression plasmid. Cells were imaged by video microscopy 32 h post transfection for 24 h in 5 min intervals. Depicted are images of a L2-GFP expressing cell progressing through mitosis (from Video S9). (D) Timing of L2-GFP chromosomal association relative to metaphase plate formation (n = 88).

If the subviral DNA/L2 complex gains access to the nucleoplasm after NEB, the question remained of how the complexes ensure incorporation into the newly forming daughter nuclei. To answer this question, we analyzed the localization of EdU-labeled vDNA in mitotic cells after infection of unperturbed cells with EdU-HPV16. During mitotic progression the vDNA spots were found to be increasingly associated with condensed host cell chromatin (Figure 5A, B). The association increased from prophase over prometaphase to metaphase, after which no further increase during ana- and telophase was observed (Figure 5A, B). This suggested that the vDNA associated with the host cell chromosomes after NEB.

To analyze the dynamics of association of the subviral complex with condensed host cell chromatin during mitosis, and the contribution of L2, we expressed L2 fused to GFP and followed their intracellular localization in live HeLa H2B-mCherry cells. As expected, L2-GFP localized to the nucleus during interphase (Figure 5C, Video S9), since it is imported by karyopherin-mediated transport [67]. Upon nuclear envelope breakdown, it was dispersed throughout the cytosol (Figure 5C, Video S9). Upon metaphase plate formation, L2-GFP associated with host cell chromatin, and remained associated with it throughout cytokinesis (Figure 5C, Video S9). These findings supported a model in which the association of the subviral complex with condensed host cell chromatin was mediated by L2. Since a visible L2-GFP association with host cell chromatin was observed almost exclusively after metaphase plate formation, i.e. mostly within 5–15 min post metaphase plate formation (Figure 5D), it is likely that the subviral complex is recruited to host cell chromatin specifically during this time window.

### The timing of NE absence is rate-limiting for infection

These findings explained, why HPV16 nuclear entry was blocked until mitosis occurred during cell cycle progression. However, the question remained why RNAi-mediated silencing of certain mitosis regulators would enhance infectivity of HPV16.

To analyze whether any particular mitotic phenotype may correlate with enhanced infection, we depleted some of the mitotic regulators that enhanced HPV infection or, as controls, genes that reduced infection. We examined this in HeLa cells expressing H2B-mRFP and IBB-GFP, and determined whether defects in mitosis could be observed by live cell microscopy.

**Figure 6:**
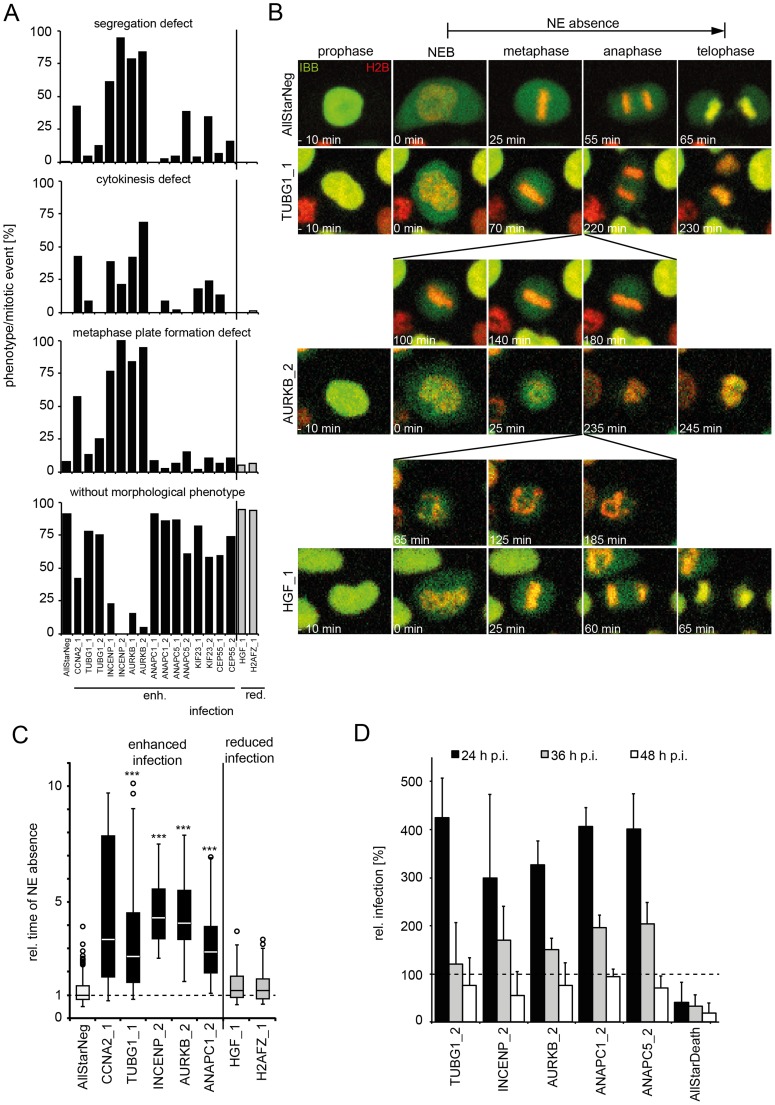
Mitotic regulators that enhance HPV16 infection exhibit prolonged mitoses upon RNAi. HeLa H2B-mRFP/IBB-GFP cells were reverse transfected with the indicated siRNAs (see also Table S4). (A) Cells were imaged by video microscopy 48 h post transfection for 24 h in 5 min intervals. Mitotic events after RNAi were assigned to one or more morphological phenotypes such as defects in metaphase plate formation, chromosome segregation, cytokinesis, or designated without morphological phenotype (from top to bottom). (B) Time-lapse images of representative cells from (A). Mitotic progression is depicted for cells after RNAi of TUBG1 (2nd row, see Video S11), AURKB (3rd row, see Video S12), HGF (4th row, see Video S13) or control (AllStar Neg., 1st row, see Video S10). (C) The timing from NEB until telophase (i.e. nuclear accumulation of IBB-GFP) was used to define the time of NE absence. Relative times of NE absence are presented as boxplots with outliers (circles). Statistical significance was determined by a one-tailed, independent, heteroscedastic t-test; ***: p-value <0.001. (D) 48 h after reverse transfection of indicated siRNAs, cells were infected with HPV16-GFP, and infection was scored at 24 h, 36 h, and 48 hpi by automated microscopy and computational image analysis. Depicted is the infection relative to AllStarNeg control in percent ± SD.

A variety of mitotic phenotypes were observed including defects in metaphase plate formation, in chromosome segregation, and in cytokinesis (Figure 6A), but entry into mitosis and NEB readily occurred (Figure 6A–C, Table S3). These phenotypes did not correlate with the effect on infection. The only correlation with infection was the duration of the time window during which the NE was absent. Prolonged NE absence always correlated with enhanced infection (Figure 6C, Table S3, Videos S10–14, compare Table S1). In fact, consistent with prolonged mitosis, RNAi of genes that enhanced infection resulted on average in a significantly increased mitotic index (≥1.5 z-scores) over those that reduced infection (Figure S6). This was supported by additional observations: First, careful analysis of live cell videos of individual monastrol-treated cells showed that the longer mitosis lasted, the earlier GFP expression from HPV16 infection could be detected, following completion of the mitosis (Figure S7A). Secondly, when examining cell divisions with and without added virus, we observed that, although HPV16 infection did not affect the number of mitoses per cell number, it did increase the percentage of prolonged mitosis events, 9 versus 27% (Figure S7B–E). Third, expression of L2-GFP induced a prolongation of mitosis (Figure S7F, compare Video S14, 15). The duration of mitosis increased with higher amounts of L2-GFP signal (Figure S7F). If higher amounts of L2-GFP were present in cells (i.e. ≥100% signal above background), these cells entered mitosis, but failed to divide, and subsequently became apoptotic, indicating that the checkpoint was not overcome (Video S16). Taken together, these observations indicated that the time of NE absence was rate-limiting for infection and nuclear import of the vDNA, and that association of L2 with host cell chromatin was able to increase the length of mitosis thus favoring nuclear import.

To test whether prolongation of mitoses enhanced the probability of nuclear import (and infection), we depleted cells of mitotic regulators from the screen such as AURKB, INCENP, and ANAPC by RNAi to induce prolonged mitosis. They were then infected for 24, 36, and 48 h to cover the time when the first cells expressed GFP to the time when the number of GFP expressing cells reached maximum [31]. Strikingly, the infectivity was strongly enhanced at 24 h p.i. in cells depleted of the mitotic regulators, whereas the effects at 48 h p.i. were minor (Figure 6D). These data were consistent with a higher probability of vDNA localization to the nucleoplasm if the time window without an intact NE was longer.

To verify our hypothesis, we depleted two additional genes by RNAi that prolong mitosis according to published work (the mitotic phosphatase PPP2R2A and the centromere protein CENPE, [68], [69]). As expected, depletion of PPP2R2A and CENPE led to prolongation of mitosis (Figure S8A, Table S3, S4). When PPP2R2A- and CENPE -depleted cells were infected with HPV16-GFP, infection was increased (Figure S8B), which supported the notion that the length of NE absence may be a kinetic bottleneck for import of the vDNA during mitosis.

## Discussion

To screen for cell factors that influence entry and other early events in the HPV infection cycle, we employed a two-step strategy with a total of six siRNAs per gene. We relied on a robust, image-based infection assay, supervised classification of phenotypes and an improved bioinformatic pipeline [41], [45]. To avoid some of the pitfalls inherent in the RNAi approach [40], we focused our validation on gene clusters rather than individual hits.

The primary screen involved 7000 human genes targeted in the so-called druggable library of siRNAs (Qiagen). Of the 315 potential hits, 291 were followed up in a secondary screen with additional siRNAs, and 162 could be confirmed. Our confidence in the results was strengthened by the identification of several factors previously described to be involved in HPV16 infection. The results of the follow-up studies demonstrated that not only genes that give reduced infectivity after silencing can lead to novel insights but also those hits that show enhanced infection.

A recent genome-wide RNAi-screen for HPV16 PsV infection in HeLa-S3 cells revealed the importance of retrograde membrane trafficking to the Golgi complex and a role for the retromer as an important determinant in HPV16 entry into cells [34]. Out of the 261 and 162 hits identified by Lipovsky et al. and by us, respectively, four were common (Figure S9). This low number of overlapping genes is not unusual when RNAi screens are compared, and the overlap typically increases significantly, when clusters of genes are compared [40], [41], [70]. This was also the case for the two HPV screens when significantly enriched functional annotation clusters were compared (Figure S9). For example, both studies identified the importance of a cluster associated with the Golgi complex. A more thorough comparison was not possible, because the results of the Lipovsky screen are only partially published.

Besides the Golgi cluster, the bioinformatic analysis of our screen revealed a number of clusters with multiple hits associated with known pathways or complexes. The most prominent was a set of genes involved in the regulation of mitosis and the cell cycle. This cluster has not been observed in screens performed with other viruses by us or published by others ([40], [45], [71], unpublished data), and may be unique to papillomaviruses. Most of the genes in the cluster were inhibitory, i.e. they showed enhanced infection after silencing suggesting that they function to limit infection. Moreover, it was striking that the hit list failed to include nuclear import factors or nuclear pore components that have been seen as prominent hits in screens against other viruses including those that replicate in the nucleus [40], [45]. This suggested that, unlike these other viruses, HPV may not be using the nuclear pores and nuclear transport receptors for accessing the nucleus during the host cell entry.

Our results showed that the virus was efficiently internalized by endocytosis, routed to the perinuclear area, and partially uncoated in interphase cells. However, the viral DNA failed to enter the nucleus. Therefore no transcription of the marker protein GFP occurred. Mitosis was the key step that allowed infection to proceed. The expression of GFP from the vDNA correlated perfectly with mitosis; the GFP became detectable 4.5±0.4 h after mitosis was completed. Moreover, since late steps in mitosis such as chromosome separation and cytokinesis were not required, the critical process could be narrowed down to one of the early steps.

That the critical step was the disruption of the NE was indicated by two observations. First, co-infection experiments in interphase cells showed that transient NE perforation by parvovirus H1 allowed HPV infection. This implied that the viruses were able to reach a stage in their entry program where they were ready to enter the nucleus productively, but could only do so if the NE integrity was compromised. Secondly, analysis of some of the hits from the siRNA screen indicated that mitosis provided a kinetic bottleneck for the nuclear import of viral genomes. The average time during mitosis when the NE is disrupted is about 45–60 min in HeLa cells. After depletion of INCENP, ANAPC, and AURKB, the time window was extended 3–5 fold resulting in a doubling or more in infection. This suggested that the longer the period without a functional membrane barrier, the better the chances for the virus to be productively included when daughter nuclei were formed. We also noted that while the addition of HPV16 itself did not promote division in the cell population, it did cause a significant increase in the fraction of cells that displayed prolonged mitosis, a process that appears to depend on the amount of L2 associating with host cell chromatin. This may be a mechanism for the virus to promote vDNA import into nuclei, a difficult step in the entry program. The results were consistent with observations of Pyeon and coworkers, who showed that cell cycle progression into early mitosis is required for HPV16 PsV infection [39]. Our data allowed us to define the key step as the breakdown of the NE barrier. Since raft-derived HPV16, as well as HPV6 and 18 PsV exhibted similar requirements, it is likely that cell cycle progression into early mitosis represents a general requirement for papillomavirus infection.

We propose that after virus internalization, trafficking, conformational alterations, and penetration into the cytosol, L2/vDNA complexes wait in the perinuclear space until NEB occurs. When the nuclear components are rendered accessible, the subviral complexes associate with the condensed chromatin, and are included in newly forming daughter cell nuclei. Such a mechanism has not been observed for other viruses that replicate in the nucleus except for certain retroviruses that also depend on mitosis for provirus integration [57], [63]. Most other DNA viruses make use of the nuclear import machinery and the nuclear pore complexes.

The inclusion of the HPV16 genome into the newly forming nuclei involves the participation of the minor capsid protein L2, since it is the only viral protein that accompanies the vDNA into the nucleus [17], [35], [38]. L2 contains two functional nuclear localization signals (NLSs) [67].While these are necessary for nuclear import during virus assembly, they are hardly important for nuclear inclusion during virus entry. In fact, the N-terminal NLS is cleaved off by the cellular protein convertase furin in an earlier stage of entry [11], [17], [18]. The C-terminal NLS is involved in binding both to dynein light chains and to the vDNA [43], [72], [73]. Strikingly, L2 associated with condensed host cell chromatin on the metaphase plate, which may indicate an active recruitment of the subviral complex during mitosis to ensure enclosure of the vDNA into newly forming nuclei. It is tempting to speculate that L2 interacts specifically with cell proteins or nuclear structures that facilitate nuclear localization. Such a role has been observed for the p12 protein of murine leukemia virus and the E2 protein of HPV [74], [75]. It is interesting to note that HPV appear to encode two proteins that secure vDNA localization to daughter nuclei during cell division. Whereas E2 links the replicated, episomal vDNA to chromosomes by cellular adapter molecules during cytokinesis in suprabasal layers, L2 may fulfill a similar role during entry. The association of HPV16 vDNA with host cell chromatin and its enclosure into newly forming nuclei is efficient, since the majority of vDNA located on host chromosomes during metaphase and in the nucleus after cytokinesis.

That HPV16 entry did not induce mitosis or nuclear envelope breakdown suggested that nuclear entry of the vDNA is dependent on physiologically occurring cell divisions. This provides an explanation for the narrow cell specificity of HPV; it exclusively infects basal stem cells or transiently amplifying cells in human skin or mucosa [1]. Only these are mitotically active. One possible advantage may be that, since the transcription and replication of HPV vDNA is tightly coupled to the differentiation program of keratinocytes [76], it may be beneficial to limit viral transcription and replication during G1 phase, and thus avoid immune detection. In addition, it may be favorable to restrict productive entry to basal cells in order to provide a reservoir of persistently infected cells. The advantage may outweigh the risks that the prolonged viral entry process would trigger innate immune responses, which might lead to viral clearance during entry.

## Materials and Methods

All experimental results are derived from at least three independent experiments.

### Cell lines

HeLa cells were from ATCC. HaCaT cells that originated from Norbert Fusenig at the DKFZ, Heidelberg, Germany, were a gift from the Schiller lab (NIH, NCI, Bethesda, USA). HeLa H2B-mRFP/IBB-GFP and HeLa H2B-mCherry were a gift of D. Gerlich, Vienna [48]. HeLa IBB-EGFP cells were obtained after transfection of HeLa Kyoto cells with pIBB-EGFP [65] and clonal selection with 500 µg/ml G418 (PAA). Cells were maintained in DMEM (Gibco) supplemented with 10% fetal calf serum (Biochrom). Medium for stable cell lines was supplemented with 500 µg/ml G418. For HeLa H2B-mRFP/IBB-GFP cells 0.5 µg/ml puromycin was additionally added. PHK cells were from PromoCell.

### Viruses

Viral preparations have been previously described for HPV16, 18 and 6 PsV using plasmids pCIneo, pRwB and p16SheLL, p18sheLL [77], [78], and p6sheLL [79], respectively, for HPV16 PsV containing BrdU-labelled DNA (BrdU-HPV16) [38], for HPV16 PsV containing EdU-labelled DNA (EdU-HPV16) [37], for fluorophore labelled HPV16 PsV [80], for recombinant HSV-1 expressing GFP under control of the CMV promoter (HSV-1-GFP) [61], and for parvovirus H1 [64]. Raft-derived HPV16 was generated in organotypic raft culture of human foreskin keratinocytes that harbor episomal HPV16 (114/B) genomes as previously described [81]. In brief, raft tissues were grown on a collagen matrix for 20 days, and subsequently subjected to dounce homogenization. The homogenate was benzonase-treated, after which the homogenate was cleared of cell debris by differential centrifugation [82].

### Reagents and antibodies

Hoechst 33258 was from Invitrogen, RedDot2 was from Biotium, and all other reagents were from Sigma. The L1-7 monoclonal antibody was a gift of M. Sapp, Shreveport [54]. The TGN46 antibody (AHP1586) was from AbD Serotec. The LAMP1 (H4A3) antibody (sc-20011) was from Santa Cruz Biotechnology. AlexaFluor labeled secondary antibodies were from Invitrogen. BrdU labeling and detection kit I was from Roche. EdU Click-iT imaging kit was from Invitrogen. siRNAs were from Qiagen. The HPV16 L2-GFP expression plasmid was a gift by M. Mueller (DKFZ, Heidelberg, Germany).

### RNAi screen

Procedures are detailed in the Text S1.

### Infection studies

1×10^5^ cells were treated for 16 h prior to infection with cell cycle inhibitors at 15 µM and 100 µM for aphidicolin or monastrol, respectively, or at indicated concentrations. Cells were infected with HPV16-GFP (1 viral genome equivalent (vge)/cell) or HSV-1-GFP (0.1 plaque forming units (PFU)/cell) to result in ≈20% infection for the control. For infection of PHK, cells were treated as above, but infected with HPV16-GFP (100 vge/cell). Cells were fixed 48 h p.i. or 6 h p.i. with 4% paraformaldehyde (PFA), and analyzed by flow cytometry as described [83]. In case of NH_4_Cl exchange for aphidicolin, cells were treated as above, but 24 h p.i., aphidicolin-containing medium was exchanged for medium with 10 mM NH_4_Cl/10 mM HEPES. Cells were fixed in 4% PFA at 48 h p.i. or post washout, and analyzed as above.

For infection with HPV6 and HPV18, 5×10^4^ HeLa cells were treated for 16 h prior to infection with 3 µM aphidicolin. Cells were infected with HPV6- and HPV18-GFP in the absence and presence of aphidicolin to result in about 20% infection for untreated control. Cells were fixed 48 h p.i. with 4% PFA, and analyzed by flow cytometry.

For infection with raft-derived HPV16, 5×10^4^ HaCaT cells were seeded 48 h prior to infection. Cells were treated with 0.2 µM aphidicolin as above. Cells were infected in the presence or absence of aphidicolin with 5 vge/cell. Total RNA was extracted from cells with the Qiagen RNeasy Mini Kit at 48 h p.i.. Infection was measured by amplification of the viral splice transcript E1∧E4 normalized to the endogenous cellular control mRNA TATA-Box binding protein (TBP) in a one-step reverse transcription quantitative PCR using the Qiagen Quantitect Probe RT-PCR Kit. Primers and Probes for HPV16 E1∧E4 and TBP were used as described [82].

For infection studies after RNAi, 2000 or 1500 HeLa cells were seeded into 96-well optical-bottom plates for HSV-1 or HPV16 infections, respectively, and cells were reverse transfected with 5 nM of indicated siRNAs. 48 h after transfection, cells were infected with HSV-1-GFP or HPV16-GFP as above, and infection was scored at 6 h or 36 hpi, respectively, by automated microscopy and computational image analysis. Cell numbers and raw infection indices for each well were determined using a MATLAB-based infection scoring procedure described previously [84]. The raw infection indices were normalized to AllStarNegative siRNA-transfected control cells to result in relative infection percentages.

For the analysis of HPV16 infection kinetics upon knockdown of mitotic regulators, 2000 HeLa Kyoto cells were seeded into optical 96-well plates and reverse transfected with 5 nM siRNAs. 48 h after transfection, cells were infected with HPV16-GFP, and infection was scored at 24 h, 36 h and 48 hpi as above.

For video microscopy of infected cells, HeLa H2B-mCherry cells were plated on optical 96-well plates (Nunc), or 8-well Labtek chambers (Nunc), and pretreated with aphidicolin for 6-16 h for synchronization. The medium was exchanged 30 min prior to infection to medium containing solvent, monastrol or aphidicolin. Cells were infected with HPV16-GFP (30 vge/cell), or HSV-1-GFP (0.1 PFU/cell).

### NE perforation

1.5×10^4^ HeLa IBB-EGFP or H2B-mCherry were plated on 8-well Labtek chambers and treated with aphidicolin for 16 h. HeLa IBB-EGFP cells were infected with parvovirus H1 (200 PFU/cell) in the presence of aphidicolin. Alternatively, HeLa IBB-EGFP or H2B-mCherry cells were infected with or with HPV16 PsV expressing dsRed or GFP (50 vge/cell), respectively, followed by infection with parvovirus H1 (200 PFU/cell) at 12 h post HPV16 addition. Subsequently, time-lapse microscopy was started.

### Video microscopy

Time-lapse images were acquired on an ImageXpress Micro (Molecular Devices) under environmental control with a CoolSnap HQ camera (Photometrics) for Videos S1–4. Alternatively, a Zeiss Axiovert Z.1 automated live cell microscope, equipped with a spinning-disk-head (Yokogawa), and a CoolSnap HQ camera was employed for image acquisition. If not otherwise stated, images from infection studies were acquired every 10 min for 48 h (HPV16) or 24 h (HSV-1, H1), with a 10× or 20× objective. For phenotyping of RNAi-mediated perturbation of mitosis and L2-GFP localization during mitosis, images were acquired every 5 min for 24 h total with a 20× objective.

### Immunofluorescence analysis

To visualize vDNA using BrdU-HPV16, 3×10^4^ HeLa H2B-mCherry cells were seeded on coverslips, and treated with aphidicolin 16 h prior to infection. Cells were infected with BrdU-HPV16 (500–1000 vge/cell) in the presence or absence of aphidicolin. Cells were fixed 20–24 h p.i. in 70% ethanol containing 15 mM glycine. For detection, the BrdU Labeling and Detection Kit I (Roche) was used according to the manufacturer's instructions, except that anti-mouse AlexaFluor488 was used as secondary antibody. In case of EdU-HPV16, HeLa Kyoto cells were used, cells were fixed in 4% PFA, and for detection the Click-iT EdU imaging kit (Invitrogen) was used. After the EdU-Click-iT reaction, cells were immunostained with TGN46 and LAMP1 antibodies.

To analyze virus trafficking and partial uncoating, 3×10^4^ HeLa cells were seeded on coverslips, treated for 16 h with aphidicolin, and infected with HPV16 PsV (50–100 vge/cell) in the presence or absence of aphidicolin. Cells were fixed 16 hpi in 4% PFA, and immunostained with anti-L1 antibody (L1-7), Atto-488 phalloidin, and RedDot2.

Cells were imaged using a Zeiss LSM 510 (BrdU-HPV16) or LSM 780 (L1-7, TGN46, LAMP1 stainings; EdU-HPV16) confocal microscope. For presentation, images were adjusted and assembled using Adobe Photoshop CS6.

### HPV16 L2-GFP expression

5000 HeLa H2B-mCherry cells were seeded into optical 96-well plates (Nunc) and transfected with 125 ng HPV16 L2-GFP expression plasmid using Lipofectamine2000 (Invitrogen). About 30–32 h post transfection, video microscopy was started.

### Mitotic phenotyping after RNAi

2000 HeLa H2B-mRFP/IBB-GFP cells were seeded into optical 96-well plates (Nunc) and reverse transfected with 5–10 nM siRNA oligos (Qiagen, Table S4) using Lipofectamine RNAiMax (Invitrogen). About 48 h after transfection, video microscopy was started.

### Image analysis

For image analysis of mitosis length, we used NIH ImageJ 1.44j. Mitosis start and end frames were defined by visual detection of chromosome condensation and decondensation. GFP onset was defined as 10% signal over background. vDNA positive nuclei were quantified by thresholding of BrdU signals, with BrdU positivity ≥10× the signal of uninfected cells. For quantification of L1-7 and BrdU signals, images were analyzed using the Imaris (v7.2 and v7.6.4, respectively) Cell module. For segmentation and distance measurements, cell outlines were defined by F-actin and nuclear signals. L1-7 spot locations were segmented by thresholding, whereas signal intensity measurements occurred in arbitrary units compared to control. For quantification of nuclear import of IBB-GFP, Hoechst-stained nuclei were segmented by CellProfiler (v 2.0, [85], [86]). IBB-GFP mean fluorescence intensities (fluorescence intensity/nuclear area) were measured as arbitrary units. For mitotic timing of L2-GFP expressing cells, dispersal of L2-GFP marked the onset of mitosis with NEB and chromosome decondensation during telophase the end of mitosis. L2-GFP expression levels were classified according to fluorescence intensity in interphase cells (mean intensity of nuclear L2-GFP at 25 min before NEB). Fluorescence intensities ≥20% above background (mock-transfected cells) were considered as L2-GFP expression. L2-GFP chromosomal association was assessed visually by accumulating signal on chromosomes. Colocalization of vDNA of EdU-HPV16 with host chromatin or LAMP1 and TGN46 was quantified by using the Imaris Colocalization module (v7.6.4). Cytosolic EdU-HPV16 was considered as virus that did not localize either to TGN46 or LAMP1. For mitotic phenotyping, time-lapse recordings were visually inspected and mitotic events (i.e. cytosolic dispersal followed by nuclear accumulation of IBB-GFP signal) were assigned to one or more of the following phenotypes: metaphase plate formation defect, chromosomal segregation defect, cytokinesis defect, without phenotype. For mitotic timing, dispersal of IBB-GFP marked the onset of mitosis with NEB and nuclear accumulation of IBB-GFP during telophase the end of mitosis [68].

## Supporting Information

Figure S1 **Microscopic images of the primary RNAi screen.** Representative images from the primary RNAi screen with nuclei in blue (DAPI) and infected cells in green (GFP). Displayed are images of the control, after RNAi of HRAS and SLC9A8 with reduced infection (first row), and of the mitotic regulators ANAPC1, AURKB, and KIF23 with enhanced infection.
(TIF)

Figure S2 **Map of host cell factors resulting in enhanced or reduced infection in RNAi screen.** Analysis and depiction of hits from the siRNA with STRING/DAVID and Cytoscape (as in Figure 1B) for the hits separated by enhanced (A) and reduced (B) infection after RNAi of the respective gene. Mitosis regulators are highlighted in red. Genes previously implicated in HPV16 entry are highlighted in blue.
(TIF)

Figure S3 **Perturbation of cell cycle progression by small compound inhibitors.** HeLa cells were treated for 24 h with the indicated concentrations of cell cycle inhibitors and subsequently analyzed for the proportion of cells in certain cell cycle stages. For this, propidium iodide (PI) staining was combined with flow cytometric analysis to identify the cellular DNA content. Cell cycle phases were designated according to cellular DNA content. G1 refers to a single set of chromosomes (i.e. DNA content  =  1), G2/M to duplicated chromosomes (DNA content  =  2), and S refers to replicating chromosomes between the two states (DNA content >1 and <2). Error bars indicate SD.
(TIF)

Figure S4 **Internalization occurs in S-phase-arrested cells.** (A) HeLa cells arrested in S-phase by aphidicolin were infected with AF488-labeled HPV16 PsV for 6 h. Surface-bound PsV were then removed by protease treatment and the remaining cell-associated fluorescence (internalized virus) was measured by flow cytometry. Results are depicted as internalization relative to untreated cells in percent ± SD. (B) HeLa cells were infected with HPV16-GFP in the presence of aphidicolin, and the drug was replaced at 24 h p.i. by NH_4_Cl to block acid-activation of viruses. Cells were fixed 48 h after washout and analyzed for GFP expression by flow cytometry. Given are infected cells relative to untreated cells in percent ± SD.
(TIF)

Figure S5 **Nuclear import occurs in S-phase-arrested cells.** (A) To analyze nuclear import of vDNA in the presence of inhibitors, HeLa H2B-mCherry cells arrested in S-phase were infected with recombinant HSV-1-GFP (0.1 PFU/cell). Images were acquired by time-lapse microscopy in 10 min intervals after infection. Indicated is the time after image acquisition started, and after aphidicolin addition (in brackets). (B) HeLa cells treated with aphidicolin or monastrol at indicated concentrations were infected with HSV-1- GFP (0.1 PFU/cell, see also Video S4). Given is the amount of infected cells relative to solvent treated control ± SD of three independent experiments. (C) To analyze nucleo-cytoplasmic shuttling by cellular importins in S-phase-arrested cells, HeLa IBB-GFP cells were treated overnight with cycloheximide (to prevent IBB-GFP expression) and with aphidicolin. Subsequently cycloheximide was washed out (0 h), and cells were cultivated in the presence or absence of aphidicolin for 10 h to allow IBB-GFP expression and nuclear import. Depicted are microscopic images of a representative cell for each condition. (D) Quantification of nuclear IBB-GFP from (C). The intensity of IBB-GFP within the nucleus was quantified using ImageJ whereby the nuclear area was based on the Hoechst stain. The signals were normalized to cells without aphidicolin, and are depicted as relative nuclear import ± SD. (E) HeLa cells were transfected with three different siRNAs targeting NUP153 or with control siRNA (AllStarNeg). Cells were infected 48 h post transfection with HSV-1 or HPV16 PsV. Infection was scored at 6 h or 36 h p.i., respectively, by automated microscopy and computational image analysis. Depicted is the infection relative to AllStarNeg control in percent ± SD.
(TIF)

Figure S6 **Mitotic indices of RNAi screen.** The mitotic state of cells was determined according to nuclear morphologies at the end of the siRNA screen. Depicted is the distribution of the z-scores of mitotic indices for all confirmed genes that enhanced or reduced HPV16 infection (see Table S1). Dashed line indicates the average z-score for all genes.
(TIF)

Figure S7 **Characterization of mitoses in relation to HPV16 infection.** (A) To examine GFP onset post mitosis in relation to mitosis duration, monastrol-treated HeLa H2B-mCherry live cell infection data (as in bottom panel of Figure 2A) were evaluated as described for Figure 2B and plotted according to GFP onset and mitosis duration. Given is a linear regression of the data. (B) The number of mitotic events in HeLa H2B-mCherry cells from time-lapse series (compare Figure 2A) were assessed over a period of 48 h after infection with different amounts of HPV16-GFP and compared to uninfected cells imaged in parallel. Data was normalized to uninfected cells and is displayed as percent (of uninfected) ± SD. (C) To evaluate potential effects on the timing of mitoses after HPV16 infection, the mitotic events in HPV16 infected (30 PsV/cell, n = 305) or uninfected (mock, n = 314) HeLa H2B-mCherry time-lapse series were plotted according to the onset of mitosis after movie acquisition was started (i.e. addition of virus for infected cells) and duration of mitosis. Duration of mitosis was defined as the time between mitosis onset and end, i.e. chromosome condensation and decondensation respectively. The median duration of mitosis for uninfected cells was about 45 min and is indicated by a straight line. Prolonged mitoses were defined as an at least two-fold increase in mitotic duration, i.e. 90 min. (D) As in (C). The distribution of mitosis lengths is depicted for mock-infected cells, and cells infected with 30 or 300 particles per cell. (E) As in (C). The percentage of prolonged mitoses, i.e. ≥90 min, are shown for mock-infected cells, and cells infected with 30 or 300 particles per cell. Statistical significance was determined by a one-tailed, independent t-test: P-values *<0.05; ***<0.001. (F) HeLa H2B-mCherry cells were transiently transfected with an L2-GFP expression plasmid. Cells were imaged by video microscopy 32 h post transfection for 24 h in 5 min intervals. The timing from NEB (L2-GFP cytosolic dispersion) until telophase (chromosome decondensation) was used to define the time of NE absence. L2-GFP expression was categorized according to fluorescence intensity above background in interphase (0%: n = 40; 20–50%: n = 54; 50–95%: n = 29). Relative times of NE absence are presented as boxplots with outliers (circles). Statistical significance was determined by a one-tailed, independent t-test: P-value ***<0.001.
(TIF)

Figure S8 **Mitotic regulators that exhibit prolonged mitoses upon RNAi enhance HPV16 infection.** HeLa H2B-mRFP/IBB-GFP cells were reverse transfected with the indicated siRNAs (see also Table S4). (A) Cells were imaged by video microscopy 48 h post transfection for 24 h in 5 min intervals. The timing from NEB until telophase (i.e. nuclear accumulation of IBB-GFP) was used to define the time of NE absence. Relative times of NE absence are presented as boxplots with outliers (circles). Statistical significance was determined by a one-tailed, independent, heteroscedastic t-test; ***: p-value <0.001. (B) 48 h after reverse transfection of indicated siRNAs, cells were infected with HPV16-GFP, and infection was scored at 24 hpi by automated microscopy and computational image analysis. Depicted is the infection relative to AllStarNeg control in percent ± SD of one quadruplicate experiment.
(TIF)

Figure S9 **Comparison of HPV16 RNAi screens.** (A) Overlap of genes that reduced HPV16 infection upon RNAi between our RNAi screen and the genome wide screen of Lipovsky and colleagues (2013). (B) Overlap of common gene annotation clusters between our RNAi screen and the genome wide screen of Lipovsky and colleagues (2013) that exhibit a significant enrichment.
(TIF)

Table S1 **List of validated host factors involved in HPV16 infection.** Each of the 162 identified host factors is annotated with a link to their corresponding NCBI Gene pages, as well as their entries from several common gene and protein annotation databases, including GO, and PANTHER. The 162 factors confirmed in the rescreen (Y) are marked in green, not confirmed (N) in black, and factors not included in the secondary screen are marked red. The information is separated according to the effect on HPV16 infection upon RNAi of the respective gene, i.e. enhanced and reduced infection (Tabs 1–2).
(XLSX)

Table S2 **DAVID functional annotation clustering of validated host cell genes involved in HPV16 infection.** This table contains the functional annotation clustering results from DAVID Bioinformatics Resources 6.7 for the combined 159 host factors required for HPV16 infection. In addition the information is separated according to the effect on HPV16 infection upon RNAi of the respective gene, i.e. enhanced (31) or reduced (128) infection (Tabs 1–3). Three of the 162 confirmed factors were gene loci, and could not be included for analysis. Annotation clusters are further manually annotated corresponding to Figure 1 and Figure SS1.
(XLSX)

Table S3 **Mitotic duration after RNAi of selected genes.** HeLa H2B-mRFP/IBB-GFP cells were reverse transfected with the indicated siRNAs. Cells were imaged 48 h post transfection in 5 min intervals by video microscopy for 24 h. The time span from NEB until onset of nuclear accumulation of IBB-GFP was used to define the time of NE absence (for more information refer to material and methods). Listed are the median, minimal, and maximal times of NE absence, which were normalized to the median of AllStarNeg-transfected cells in the corresponding experiment. In addition, the outmost maximal outlier, number of analyzed mitotic events and p values (calculated by a one-tailed, independent, heteroscedastic t-test) are listed.
(DOCX)

Table S4 **List of siRNAs used for the nuclear import assay and mitotic phenotyping.** Internal siRNA names are juxtaposed with corresponding siRNA product names, gene symbols, Entrez gene IDs and applied final concentrations. Experimental verification of knockdown efficiencies is indicated by Y, i.e. yes, if knockdown efficiency >70% in HeLa cells, or N.D., i.e. not determined.
(XLSX)

Text S1 **RNAi screening procedures and further supplemental methods.** This text details the RNAi screening procedures. Further, it describes the methods for cell cycle analysis, the HPV16 internalization assay, and the IBB-GFP nuclear import assay.
(DOCX)

Video S1 **HPV-16 entry is completed with mitosis.** To kinetically relate mitosis and successful HPV16 entry, HeLa H2B-mCherry (red) cells were infected with HPV16-GFP (30 vge/cell, green). Time-lapse images were acquired in 10 min intervals. Movie plays with 24 frames/s, time stamp indicates time after virus addition.
(MOV)

Video S2 **S-phase arrest blocks HPV-16 entry.** Aphidicolin-treated HeLa H2B-mCherry cells (interphase cells) were infected with HPV16-GFP (30 vge/cell). Time-lapse images were acquired in 10 min intervals. Movie plays with 24 frames/s, time stamp indicates time after virus addition.
(MOV)

Video S3 **Entry into prometaphase is sufficient for HPV-16 entry.** HeLa H2B-mCherry cells inhibited in mitosis by treatment with monastrol were infected with HPV16-GFP (30 vge/cell). Time-lapse images were acquired in 10 min intervals. Movie plays with 18 frames/s, time stamp indicates time after virus addition. The centered cell entered prophase as seen by condensed chromosomes, but failed to align at the metaphase plate. Instead of progressing to metaphase, prometaphase was prolonged before the cell returned to G2 phase as indicated by chromosome decondensation.
(MOV)

Video S4 **HSV-1 entry and GFP expression occur irrespective of S phase inhibition.** Aphidicolin-treated HeLa H2B-mCherry cells arrested in S phase were infected with HSV-1-GFP (0.1 PFU/cell). Time-lapse images were acquired in 10 min intervals. Movie plays with 18 frames/s, time stamp indicates time after virus addition. Please note that GFP expression occurred in all cells.
(MOV)

Video S5 **IBB-EGFP serves as indicator for NEB.** Time-lapse images of HeLa IBB-GFP cells were acquired in 5 min intervals. Movie plays with 12 frames/s, time stamp indicates time after acquisition start. Note the brief cytosolic signal of IBB-GFP during mitosis.
(MOV)

Video S6 **The NE is intact during S phase inhibition.** Time-lapse images of aphidicolin-treated HeLa IBB-GFP cells were acquired in 5 min intervals. Movie plays with 12 frames/s, time stamp indicates time after acquisition start. Note the absence of any mitosis and any cytosolic signal of IBB-GFP.
(MOV)

Video S7 **Parvovirus H1 perforates the NE in S phase.** Aphdicolin-treated HeLa IBB-GFP cells arrested in S-phase were infected with parvovirus H1 (250 PFU/cell). Time-lapse images were acquired in 5 min intervals. Movie plays with 12 frames/s, time stamp indicates time after H1 addition. Please note the relocation of IBB-GFP to the cytosol of the non-dividing interphase cell.
(MOV)

Video S8 **NE perforation is sufficient for HPV-16 entry.** Aphidicolin-treated HeLa H2B-mCherry cells arrested in S-phase were infected with HPV16 (100 vge/cell) harbouring a GFP reporter plasmid for 12 h and superinfected with H1 (250 PFU/cell). Time-lapse images were acquired in 5 min intervals. Movie plays with 12 frames/s, time stamp indicates time after H1 addition. Note the GFP expression (HPV16 infection) in the non-dividing interphase cell.
(MOV)

Video S9 **L2-GFP associates with the metaphase plate.** HeLa H2B-mCherry cells were transfected with L2-GFP expression plasmid. Cells were imaged 32 h post transfection in 5 min intervals by video microscopy for 24 h. Movie plays with 3 frames/s. NEB is reflected by cytosolic dispersion of L2-GFP. Note chromosomal association of L2 after metaphase plate formation.
(MOV)

Video S10 **Mitotic progression of HeLa Kyoto cells.** HeLa H2B-mRFP/IBB-GFP cells were reverse transfected with 10 nM control AllStarNeg siRNA. Cells were imaged 48 h post transfection in 5 min intervals by video microscopy for 24 h. Movie plays with 6 frames/s, and time stamp indicates time relative to NEB in min. NE absence is reflected by cytosolic IBB-GFP signal.
(MOV)

Video S11 **TUGB1 knockdown leads to prolonged time of NE absence.** HeLa H2B-mRFP/IBB-GFP cells were reverse transfected with 10 nM TUBG1_1 siRNA (see Table S4). Video microscopy and movie features as for Video S9.
(MOV)

Video S12 **AURKB knockdown leads to prolonged time of NE absence.** HeLa H2B-mRFP/IBB-GFP cells were reverse transfected with 10 nM AURKB_2 siRNA (see Table S4). Video microscopy and movie features as for Video S9.
(MOV)

Video S13 **Mitotic progression after HGF knockdown.** HeLa H2B-mRFP/IBB-GFP cells were reverse transfected with 10 nM HGF_1 siRNA (see Table S4). Video microscopy and movie features as for Video S9.
(MOV)

Video S14 **Mitotic progression HeLa cells.** HeLa H2B-mCherry cells were mock-transfected. Video microscopy and movie features as for Video S13. Time stamp indicates time relative to NEB in min.
(MOV)

Video S15 **L2-GFP expression leads to prolonged mitosis.** HeLa H2B-mCherry cells were transfected with L2-GFP expression plasmid. Video microscopy and movie features as for Video S13. Time stamp indicates time relative to NEB in min.
(MOV)

Video S16 **High levels of L2-GFP expression lead to apoptosis.** HeLa H2B-mCherry cells were transfected with L2-GFP expression plasmid. Video microscopy and movie features as for Video S9. Time stamp indicates time relative to NEB in min.
(MOV)
