# Supplementary figures and images for: Large Scale RNAi Reveals the Requirement of Nuclear Envelope Breakdown for Nuclear Import of Human Papillomaviruses
Source: PLoS Pathog. 2014 May 29;10(5):e1004162. doi: 10.1371/journal.ppat.1004162 (PMC4038628; doi:10.1371/journal.ppat.1004162)

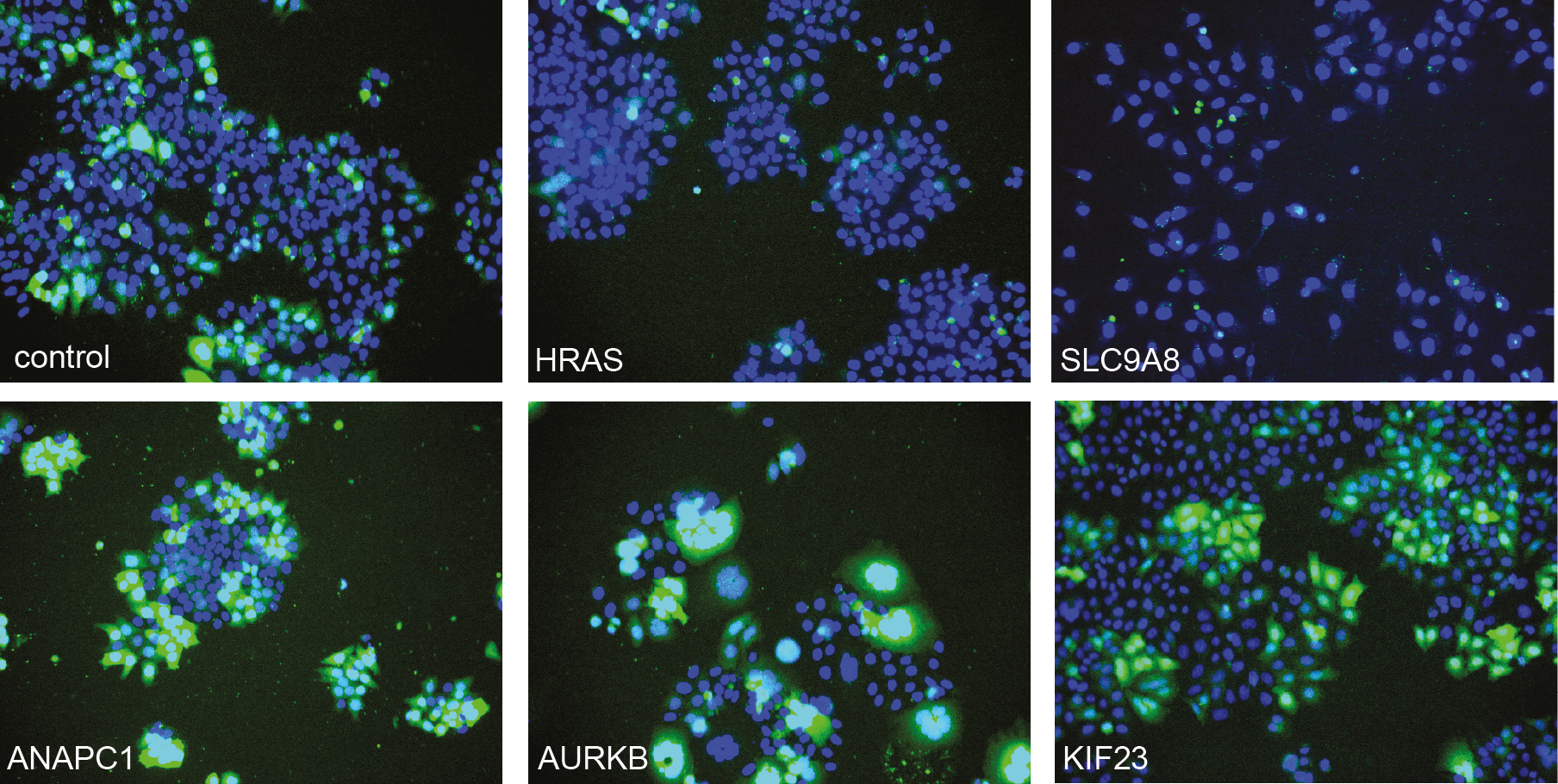

Supplement: Figure S1 — Microscopic images of the primary RNAi screen. Representative images from the primary RNAi screen with nuclei in blue (DAPI) and infected cells in green (GFP). Displayed are images of the control, after RNAi of HRAS and SLC9A8 with reduced infection (first row), and of the mitotic regulators ANAPC1, AURKB, and KIF23 with enhanced infection. (TIF) [file ppat.1004162.s001.tif]

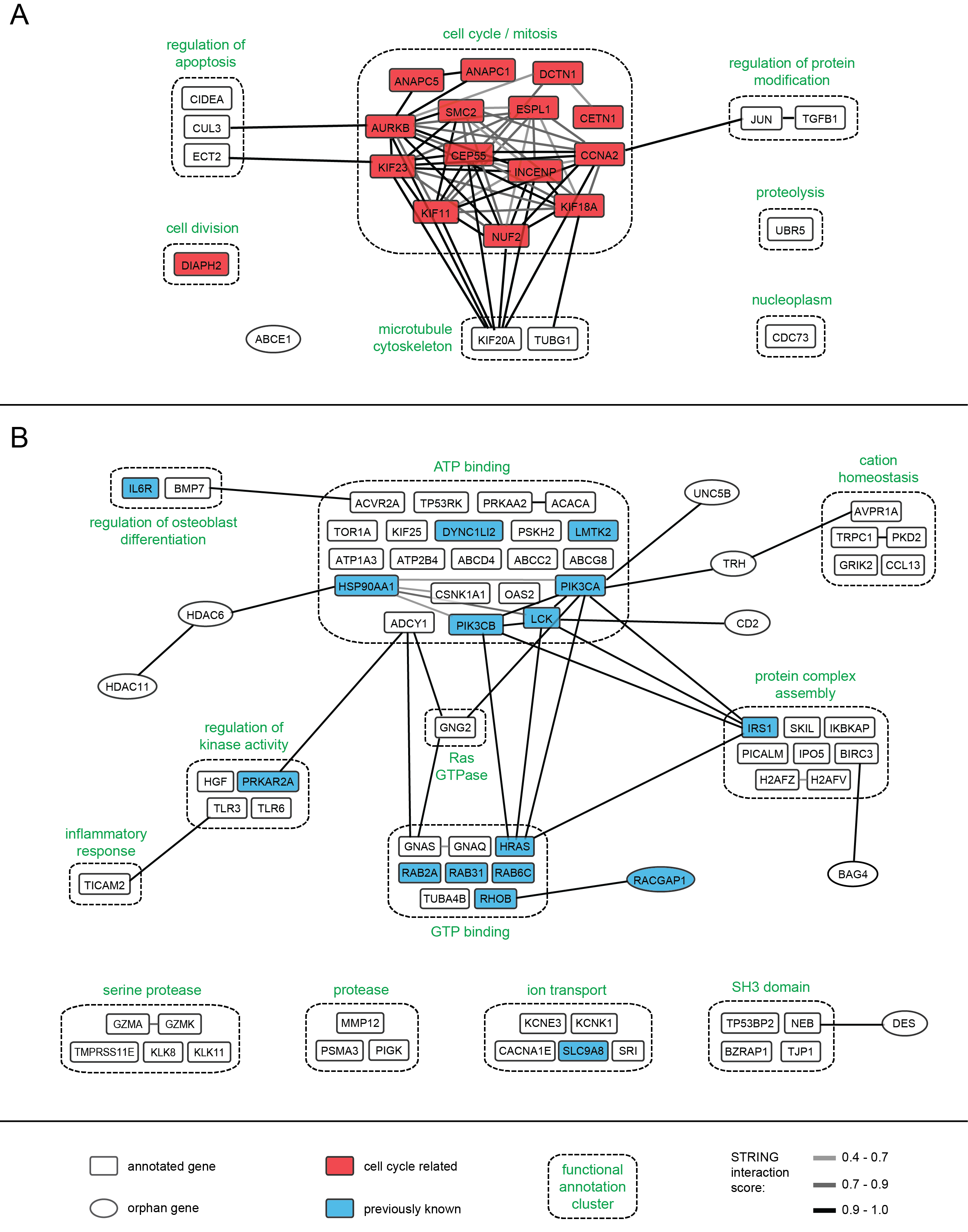

Supplement: Figure S2 — Map of host cell factors resulting in enhanced or reduced infection in RNAi screen. Analysis and depiction of hits from the siRNA with STRING/DAVID and Cytoscape (as in Figure 1B) for the hits separated by enhanced (A) and reduced (B) infection after RNAi of the respective gene. Mitosis regulators are highlighted in red. Genes previously implicated in HPV16 entry are highlighted in blue. (TIF) [file ppat.1004162.s002.tif]

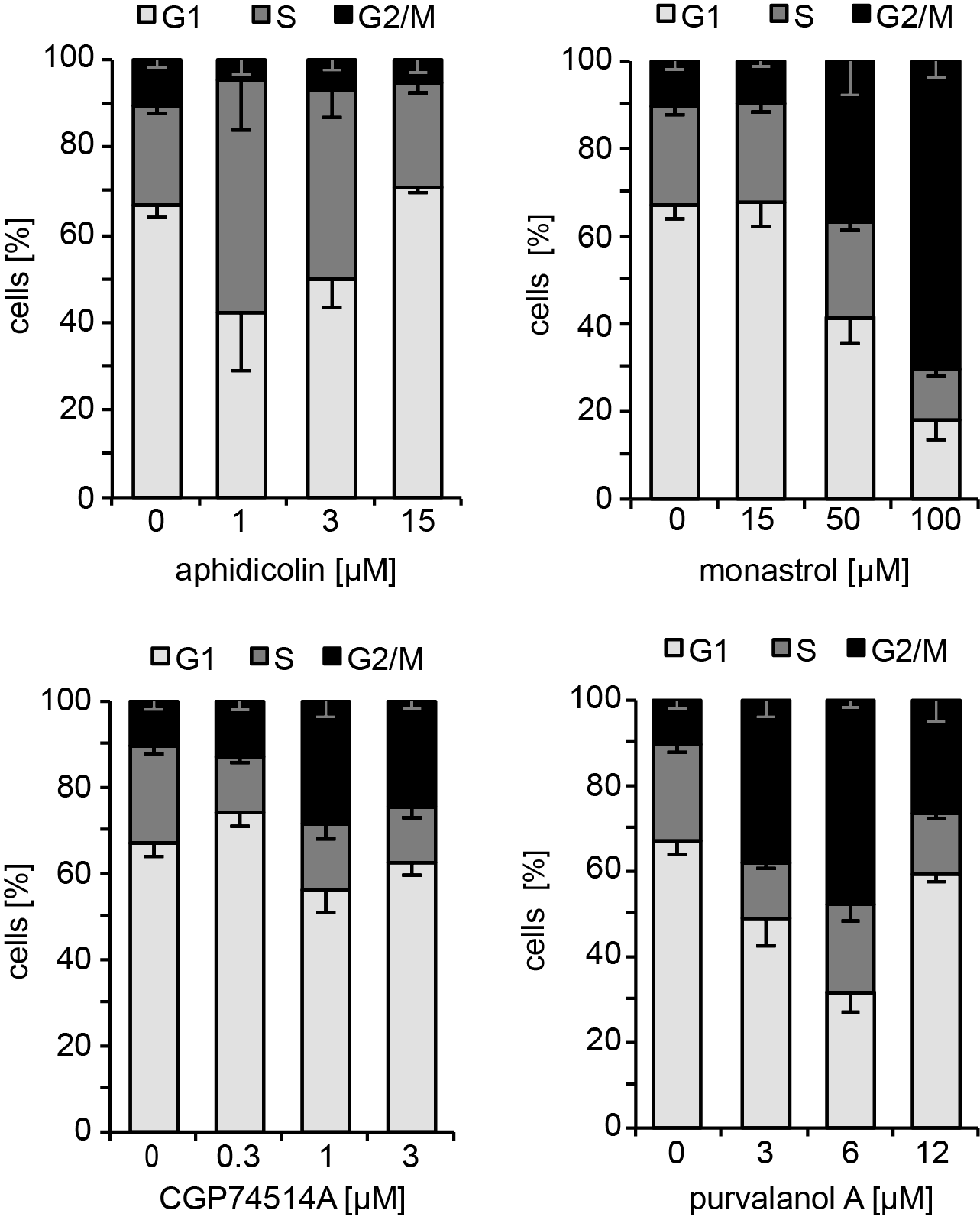

Supplement: Figure S3 — Perturbation of cell cycle progression by small compound inhibitors. HeLa cells were treated for 24 h with the indicated concentrations of cell cycle inhibitors and subsequently analyzed for the proportion of cells in certain cell cycle stages. For this, propidium iodide (PI) staining was combined with flow cytometric analysis to identify the cellular DNA content. Cell cycle phases were designated according to cellular DNA content. G1 refers to a single set of chromosomes (i.e. DNA content = 1), G2/M to duplicated chromosomes (DNA content = 2), and S refers to replicating chromosomes between the two states (DNA content >1 and <2). Error bars indicate SD. (TIF) [file ppat.1004162.s003.tif]

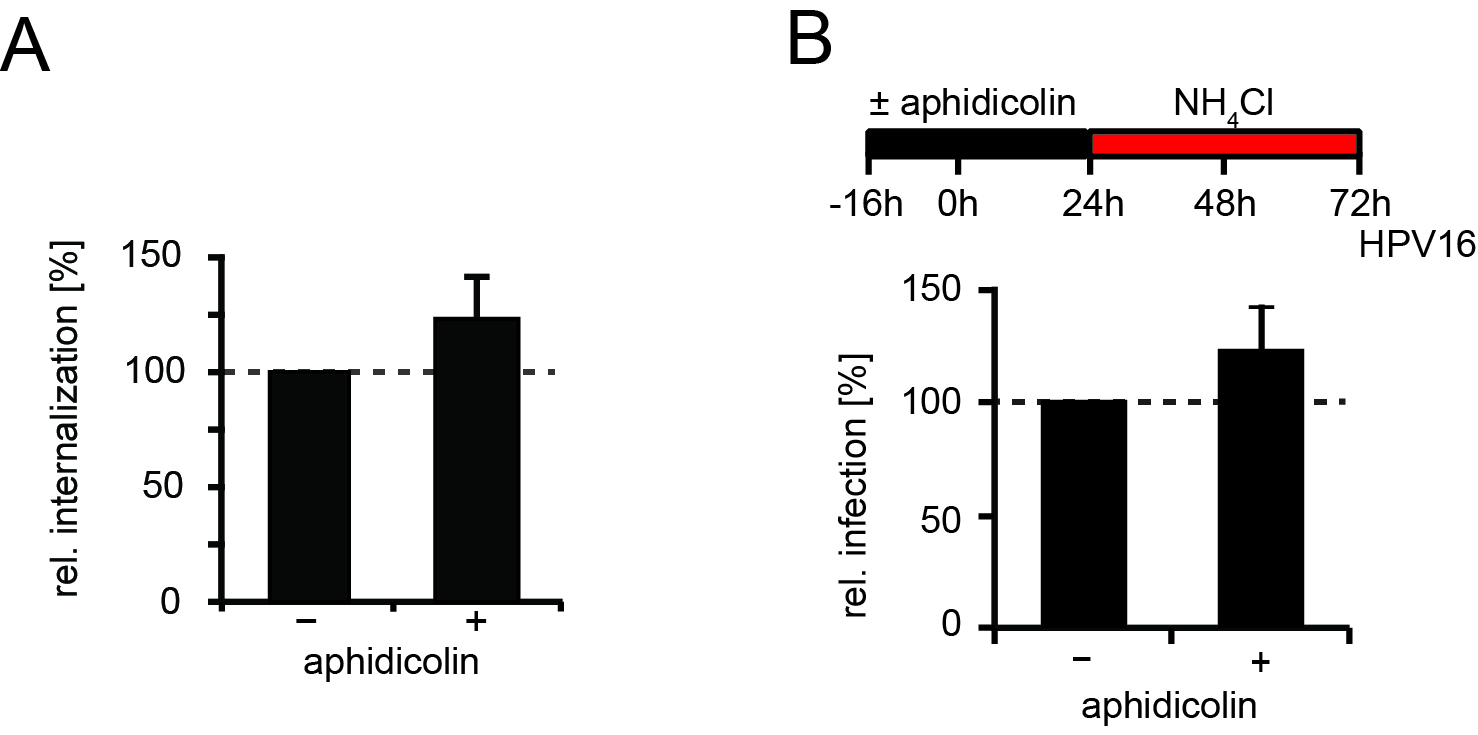

Supplement: Figure S4 — Internalization occurs in S-phase-arrested cells. (A) HeLa cells arrested in S-phase by aphidicolin were infected with AF488-labeled HPV16 PsV for 6 h. Surface-bound PsV were then removed by protease treatment and the remaining cell-associated fluorescence (internalized virus) was measured by flow cytometry. Results are depicted as internalization relative to untreated cells in percent ± SD. (B) HeLa cells were infected with HPV16-GFP in the presence of aphidicolin, and the drug was replaced at 24 h p.i. by NH4Cl to block acid-activation of viruses. Cells were fixed 48 h after washout and analyzed for GFP expression by flow cytometry. Given are infected cells relative to untreated cells in percent ± SD. (TIF) [file ppat.1004162.s004.tif]

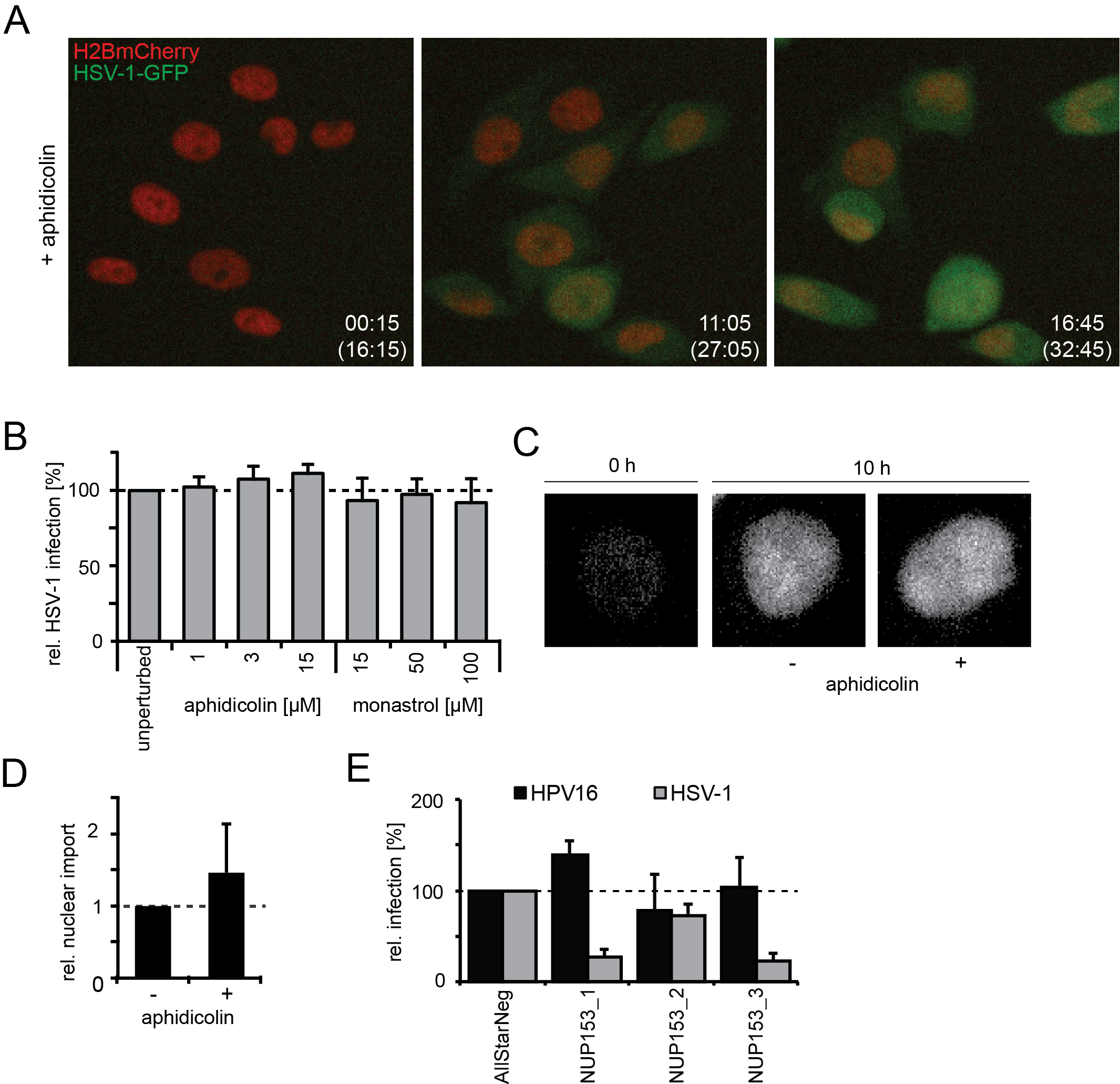

Supplement: Figure S5 — Nuclear import occurs in S-phase-arrested cells. (A) To analyze nuclear import of vDNA in the presence of inhibitors, HeLa H2B-mCherry cells arrested in S-phase were infected with recombinant HSV-1-GFP (0.1 PFU/cell). Images were acquired by time-lapse microscopy in 10 min intervals after infection. Indicated is the time after image acquisition started, and after aphidicolin addition (in brackets). (B) HeLa cells treated with aphidicolin or monastrol at indicated concentrations were infected with HSV-1- GFP (0.1 PFU/cell, see also Video S4). Given is the amount of infected cells relative to solvent treated control ± SD of three independent experiments. (C) To analyze nucleo-cytoplasmic shuttling by cellular importins in S-phase-arrested cells, HeLa IBB-GFP cells were treated overnight with cycloheximide (to prevent IBB-GFP expression) and with aphidicolin. Subsequently cycloheximide was washed out (0 h), and cells were cultivated in the presence or absence of aphidicolin for 10 h to allow IBB-GFP expression and nuclear import. Depicted are microscopic images of a representative cell for each condition. (D) Quantification of nuclear IBB-GFP from (C). The intensity of IBB-GFP within the nucleus was quantified using ImageJ whereby the nuclear area was based on the Hoechst stain. The signals were normalized to cells without aphidicolin, and are depicted as relative nuclear import ± SD. (E) HeLa cells were transfected with three different siRNAs targeting NUP153 or with control siRNA (AllStarNeg). Cells were infected 48 h post transfection with HSV-1 or HPV16 PsV. Infection was scored at 6 h or 36 h p.i., respectively, by automated microscopy and computational image analysis. Depicted is the infection relative to AllStarNeg control in percent ± SD. (TIF) [file ppat.1004162.s005.tif]

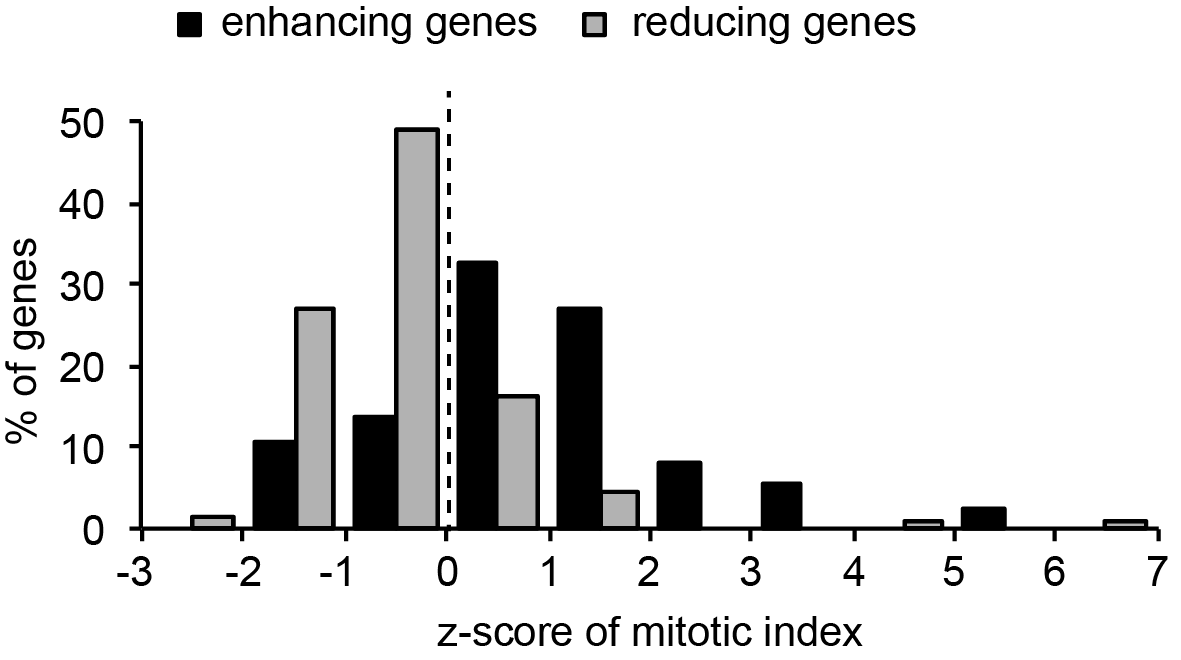

Supplement: Figure S6 — Mitotic indices of RNAi screen. The mitotic state of cells was determined according to nuclear morphologies at the end of the siRNA screen. Depicted is the distribution of the z-scores of mitotic indices for all confirmed genes that enhanced or reduced HPV16 infection (see Table S1). Dashed line indicates the average z-score for all genes. (TIF) [file ppat.1004162.s006.tif]

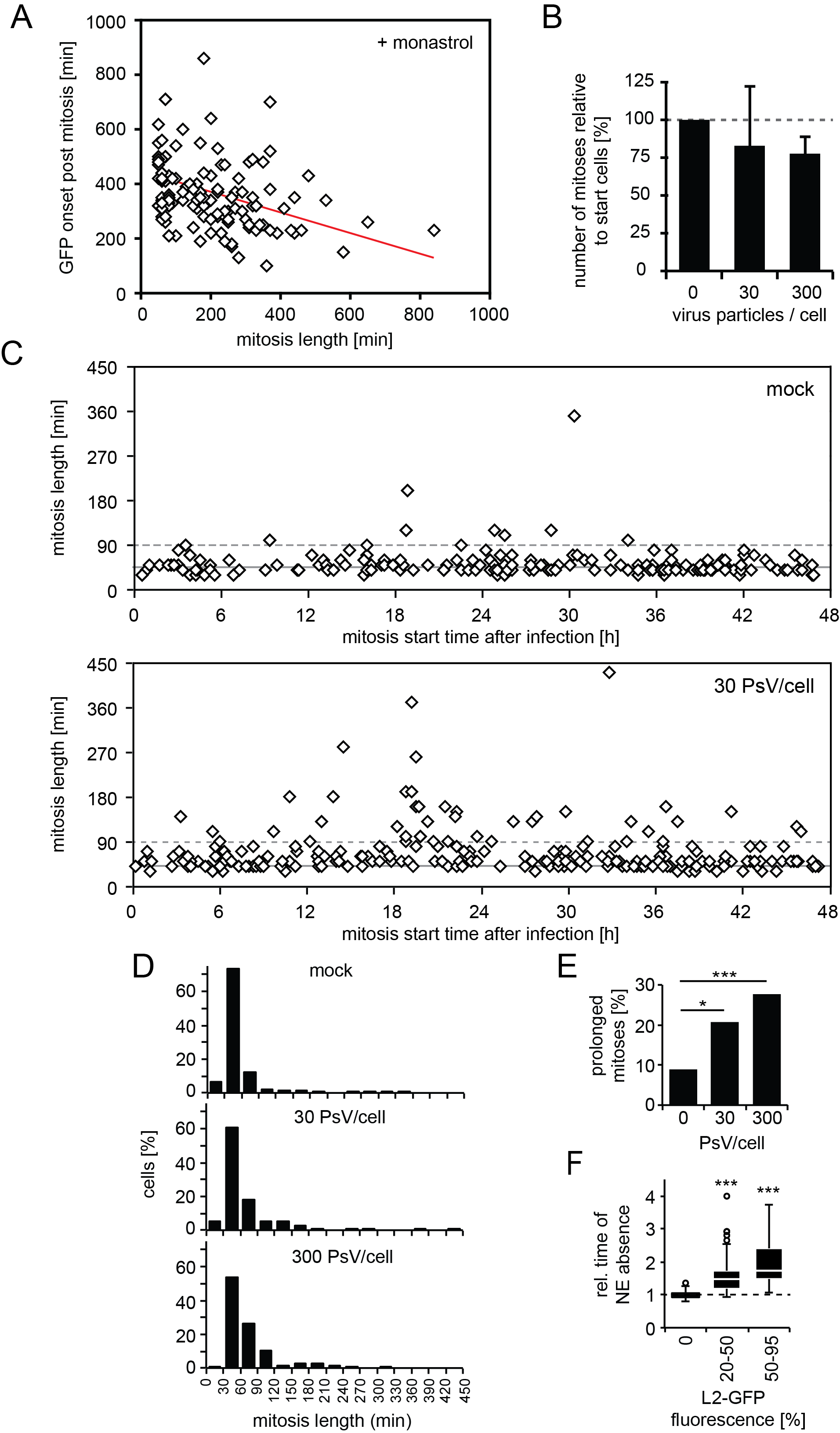

Supplement: Figure S7 — Characterization of mitoses in relation to HPV16 infection. (A) To examine GFP onset post mitosis in relation to mitosis duration, monastrol-treated HeLa H2B-mCherry live cell infection data (as in bottom panel of Figure 2A) were evaluated as described for Figure 2B and plotted according to GFP onset and mitosis duration. Given is a linear regression of the data. (B) The number of mitotic events in HeLa H2B-mCherry cells from time-lapse series (compare Figure 2A) were assessed over a period of 48 h after infection with different amounts of HPV16-GFP and compared to uninfected cells imaged in parallel. Data was normalized to uninfected cells and is displayed as percent (of uninfected) ± SD. (C) To evaluate potential effects on the timing of mitoses after HPV16 infection, the mitotic events in HPV16 infected (30 PsV/cell, n = 305) or uninfected (mock, n = 314) HeLa H2B-mCherry time-lapse series were plotted according to the onset of mitosis after movie acquisition was started (i.e. addition of virus for infected cells) and duration of mitosis. Duration of mitosis was defined as the time between mitosis onset and end, i.e. chromosome condensation and decondensation respectively. The median duration of mitosis for uninfected cells was about 45 min and is indicated by a straight line. Prolonged mitoses were defined as an at least two-fold increase in mitotic duration, i.e. 90 min. (D) As in (C). The distribution of mitosis lengths is depicted for mock-infected cells, and cells infected with 30 or 300 particles per cell. (E) As in (C). The percentage of prolonged mitoses, i.e. ≥90 min, are shown for mock-infected cells, and cells infected with 30 or 300 particles per cell. Statistical significance was determined by a one-tailed, independent t-test: P-values *<0.05; ***<0.001. (F) HeLa H2B-mCherry cells were transiently transfected with an L2-GFP expression plasmid. Cells were imaged by video microscopy 32 h post transfection for 24 h in 5 min intervals. The timing from NE [file ppat.1004162.s007.tif]

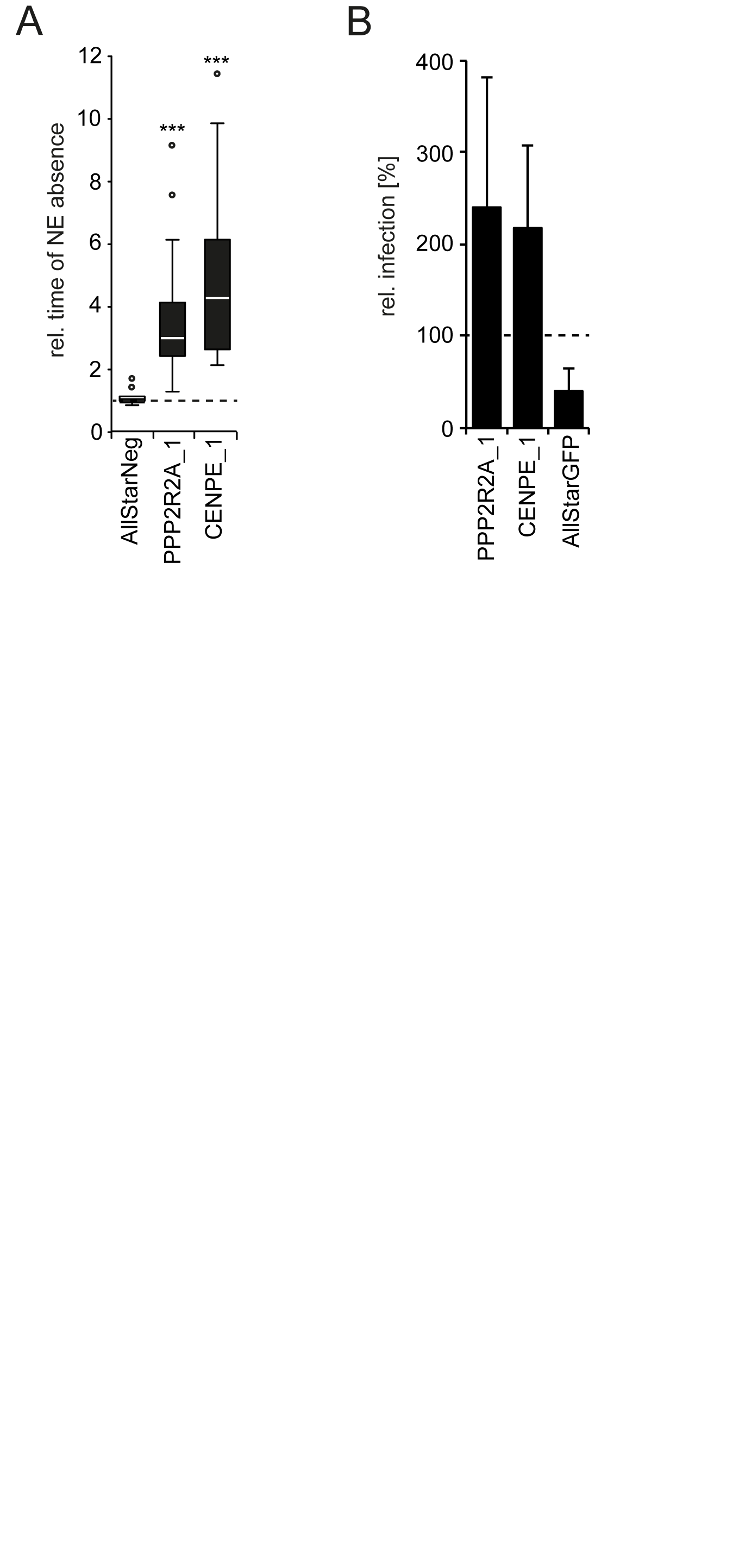

Supplement: Figure S8 — Mitotic regulators that exhibit prolonged mitoses upon RNAi enhance HPV16 infection. HeLa H2B-mRFP/IBB-GFP cells were reverse transfected with the indicated siRNAs (see also Table S4). (A) Cells were imaged by video microscopy 48 h post transfection for 24 h in 5 min intervals. The timing from NEB until telophase (i.e. nuclear accumulation of IBB-GFP) was used to define the time of NE absence. Relative times of NE absence are presented as boxplots with outliers (circles). Statistical significance was determined by a one-tailed, independent, heteroscedastic t-test; ***: p-value <0.001. (B) 48 h after reverse transfection of indicated siRNAs, cells were infected with HPV16-GFP, and infection was scored at 24 hpi by automated microscopy and computational image analysis. Depicted is the infection relative to AllStarNeg control in percent ± SD of one quadruplicate experiment. (TIF) [file ppat.1004162.s008.tif]

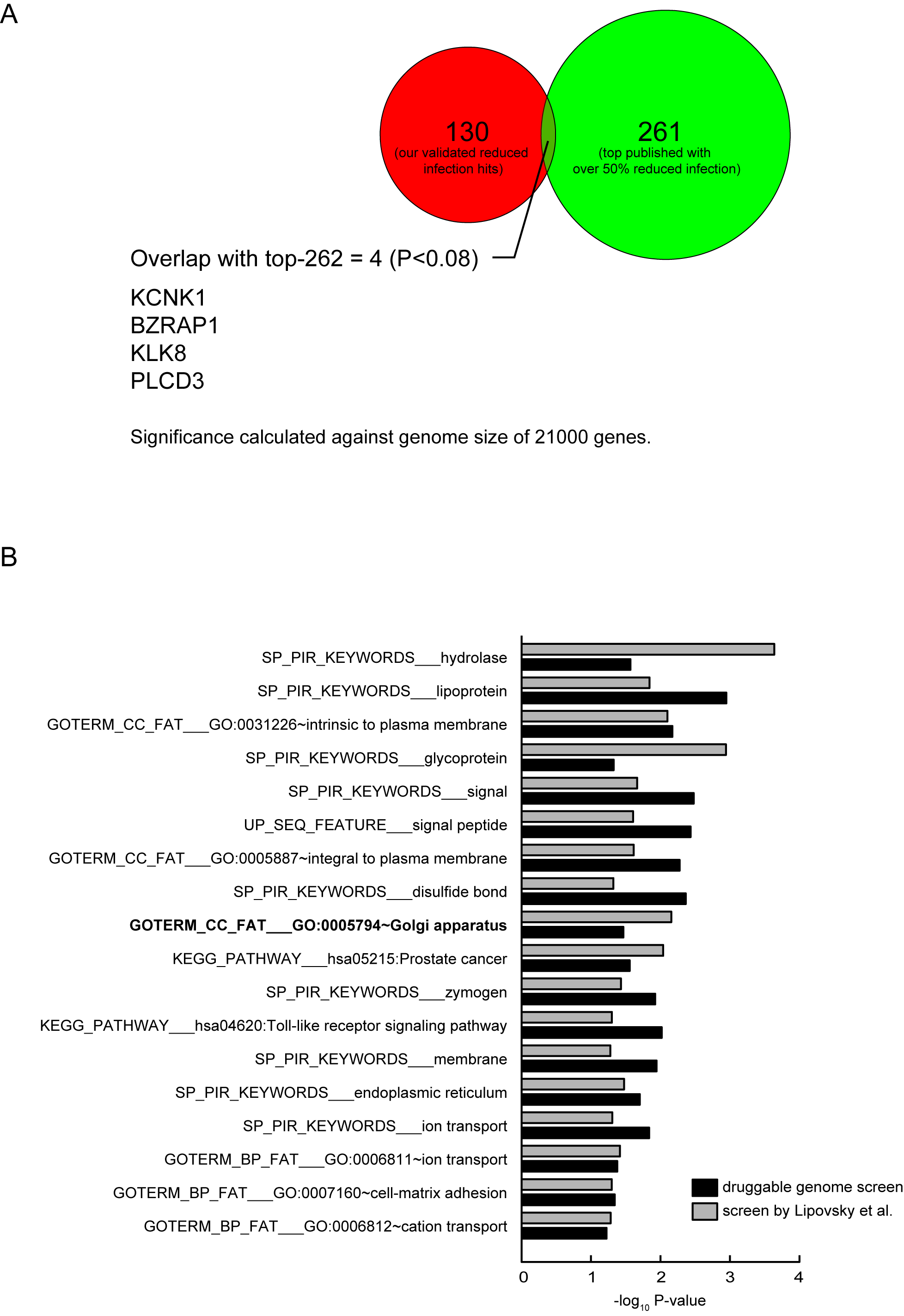

Supplement: Figure S9 — Comparison of HPV16 RNAi screens. (A) Overlap of genes that reduced HPV16 infection upon RNAi between our RNAi screen and the genome wide screen of Lipovsky and colleagues (2013). (B) Overlap of common gene annotation clusters between our RNAi screen and the genome wide screen of Lipovsky and colleagues (2013) that exhibit a significant enrichment. (TIF) [file ppat.1004162.s009.tif]
