# Supplementary material for: Large Scale RNAi Reveals the Requirement of Nuclear Envelope Breakdown for Nuclear Import of Human Papillomaviruses
Source: PLoS Pathog. 2014 May 29;10(5):e1004162. doi: 10.1371/journal.ppat.1004162 (PMC4038628; doi:10.1371/journal.ppat.1004162)
Supplement: Table S3 — Mitotic duration after RNAi of selected genes. HeLa H2B-mRFP/IBB-GFP cells were reverse transfected with the indicated siRNAs. Cells were imaged 48 h post transfection in 5 min intervals by video microscopy for 24 h. The time span from NEB until onset of nuclear accumulation of IBB-GFP was used to define the time of NE absence (for more information refer to material and methods). Listed are the median, minimal, and maximal times of NE absence, which were normalized to the median of AllStarNeg-transfected cells in the corresponding experiment. In addition, the outmost maximal outlier, number of analyzed mitotic events and p values (calculated by a one-tailed, independent, heteroscedastic t-test) are listed. (DOCX) [file ppat.1004162.s012.docx]

|  |  |  |  |  |  |  |  |
| --- | --- | --- | --- | --- | --- | --- | --- |
|  | **siRNA** | **median** | **min** | **max** | **outlier** | **n** | **p value** |
|  | AllStarNeg (Fig. 6C) | 1.00 | 0.50 | 2.26 | 3.94 | 130 |  |
|  | AllStarNeg (Fig. S9A) | 1.00 | 0.86 | 1.14 | 1.71 | 20 |  |
| enhanced  HPV16 infection | CCNA2_1 | 3.40 | 0.75 | 9.70 |  | 7 | 0.02 |
|  | TUBG1_1 | 2.63 | 0.81 | 9.03 | 10.13 | 23 | 0.0003 |
|  | TUBG1_2 | 3.50 | 1.30 | 8.63 |  | 16 | 0.0002 |
|  | INCENP_1 | 4.92 | 1.00 | 8.30 |  | 13 | 0.0001 |
|  | INCENP_2 | 4.33 | 2.58 | 7.50 |  | 19 | 2x10^-9^ |
|  | AURKB_1 | 7.90 | 3.20 | 9.30 |  | 19 | 2x10^-11^ |
|  | AURKB_2 | 4.08 | 1.58 | 7.90 |  | 19 | 2x10^-8^ |
|  | ANAPC1_1 | 1.44 | 0.69 | 2.90 | 5.00 | 45 | 0.001 |
|  | ANAPC1_2 | 2.88 | 1.06 | 6.91 | 6.94 | 35 | 7x10^-9^ |
|  | ANAPC5_1 | 0.83 | 0.50 | 2.91 | 7.13 | 46 | 0.03 |
|  | ANAPC5_2 | 2.42 | 1.25 | 9.56 |  | 13 | 0.03 |
|  | KIF11_2 | 1.33 | 0.83 | 3.29 | 5.83 | 12 | 0.1 |
|  | KIF23_1 | 1.32 | 0.58 | 2.85 | 4.10 | 50 | 0.03 |
|  | KIF23_2 | 1.38 | 0.58 | 4.33 | 7.19 | 29 | 0.01 |
|  | CEP55_1 | 1.54 | 0.75 | 3.70 |  | 30 | 0.0007 |
|  | CEP55_2 | 1.60 | 0.75 | 2.50 |  | 19 | 0.02 |
|  | PPP2R2A_1 | 4.14 | 1.29 | 6.14 | 9.14 | 26 | 6x10^-7^ |
|  | CENPE_1 | 4.29 | 2.14 | 9.86 | 11.43 | 20 | 2x10^-6^ |
| reduced HPV16 infection | HGF_1 | 1.20 | 0.58 | 3.15 | 3.75 | 57 | 0.07 |
|  | H2AFZ_1 | 1.20 | 0.60 | 3.00 | 3.40 | 79 | 0.04 |
