# Supplementary material for: Large Scale RNAi Reveals the Requirement of Nuclear Envelope Breakdown for Nuclear Import of Human Papillomaviruses
Source: PLoS Pathog. 2014 May 29;10(5):e1004162. doi: 10.1371/journal.ppat.1004162 (PMC4038628; doi:10.1371/journal.ppat.1004162)
Supplement: Text S1 — RNAi screening procedures and further supplemental methods. This text details the RNAi screening procedures. Further, it describes the methods for cell cycle analysis, the HPV16 internalization assay, and the IBB-GFP nuclear import assay. (DOCX) [file ppat.1004162.s014.docx]

**Text S1. RNAi screening procedures and further supplemental methods.**

## RNAi screen

HeLa MZ cells from Marino Zerial (MPI-CBG, Dresden) were maintained at 37°C and 5% CO_2_ in Dulbecco Modified Eagle Medium (DMEM; Gibco BRL) supplemented with 10% FCS, glutamax, and non-essential amino acids. All liquid handling steps including preparing cellplates, virus infection, and cell fixation were performed on a Freedom Evo200 liquid handling robot (Tecan) set up specifically for this purpose. Seeding, fixing and DAPI staining of the cells was performed using a Matrix Wellmate Microplate Dispenser (Thermo Scientific).

## siRNA transfection

RNAi against the 6,979 gene targets was achieved with three independent siRNAs targeting each (siRNAs #1 and #2 from Qiagen *druggable* genome version 2, and siRNA #3 from Qiagen *druggable* genome version 3). Each siRNA was tested in three independent experiments. Experiments were conducted in a 384-well plate format, amounting to 210 384-well plates. Each plate of the primary screen contained 37 mock controls (not RNAi-transfected cells), four siRNA transfection controls (RNAi against gene PLK1, see total cell number and control infection plots below; bars indicate median values, error bar 60^th^ percentile), four scrambled controls (AllStars scrambled siRNA) and 16 no-virus controls (wells to which no virus was added), see below. Almost all different siRNAs targeting the same gene were located on independent plates.

##
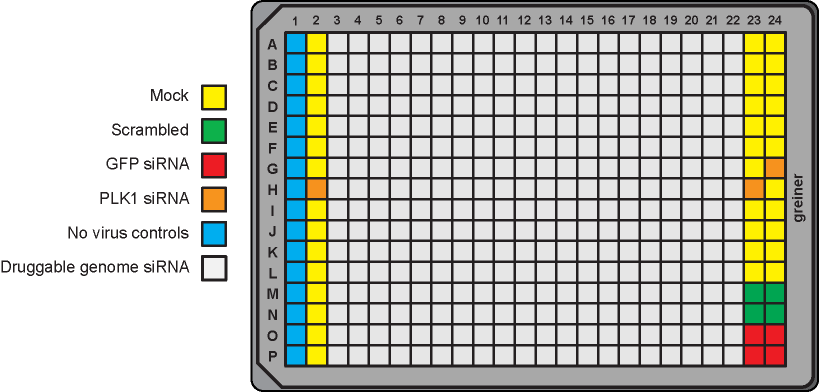

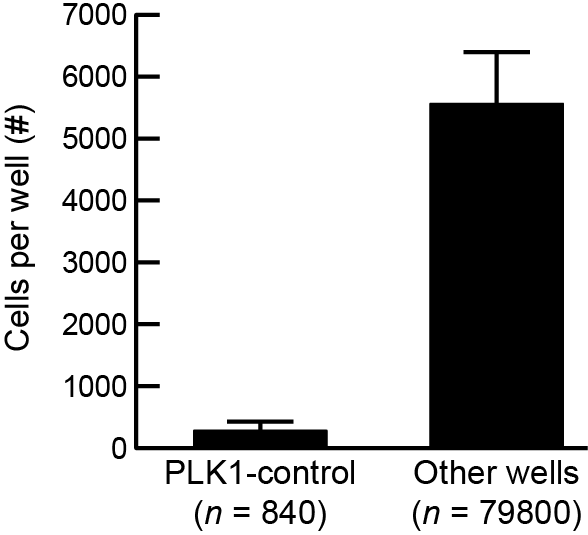

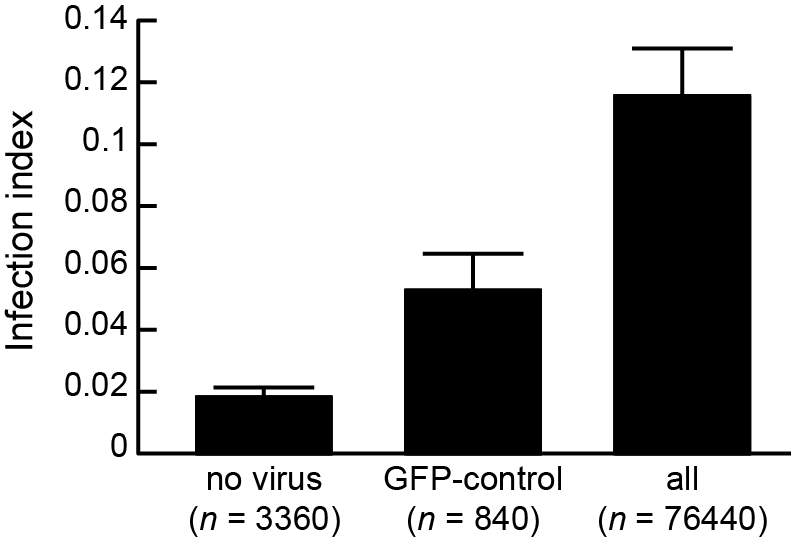


Reverse transfection of siRNA was performed by seeding the cells into a well containing 20µl of transfection mix. The transfection mix for one well contained 0.1µl LP2000 (Qiagen), 14.9µl Optimem and 5µl of 1µM siRNA (diluted in RNase free ddH2O). In each well, 900 cells were seeded in 50 µl/well of complete medium. Cells were then left at 37^o^C for 60h prior to infection.

## Infection

A single pooled HPV16 PsV stock was utilized for the entire screening procedure. A mean infection index of ~10% was chosen to allow for a dynamic range of possible up-regulating and down-regulating hits. Infection index is a combined function of the amount of virus added and the properties of the host cells [[1](#_ENREF_1)]. Therefore, the necessary amount of virus needed to achieve 10% infection was determined using “checkerboard” assays (see below).


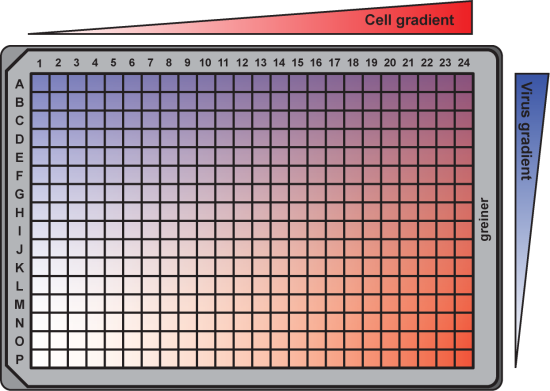


The optimal virus amount needed for ~10% infection over a wide range of cell numbers was determined by seeding a gradient of HeLa MZ cells (from 1,000 to 22,000 cells) in the columns of a 384-well plate and infecting with a gradient of virus in the rows (data not shown).

## Fixation and staining

All assays were fixed by adding PFA to a final concentration of 4%. Cells were incubated for 15-20 mins, followed by three PBS washes. To assure a homogeneous Hoechst stain, cells were permeabilized for 5 min with PBS containing 0.1% Triton X-100. After three PBS washes, the cells were incubated with PBS containing 1µg/ml Hoechst stain for 10 min. Before scanning a final wash with ddH2O was performed, this minimizes contaminants when imaging with automated microscopes.

## Imaging

All images were acquired on a 2 automated wide field cellWoRx™ microscopes (Applied Precision) with a 10× objective, and 2x2 binning per pixel. Multiwell plates were loaded onto the cellWoRx™ microscopes using Freedom Evo™ robotics from Tecan. 3×3 directly adjacent images were taken per well, covering over 85% of each well surface. An image-based auto-focus was performed on the Hoechst signal for each imaged site. The images were recorded with 12-bit CCD cameras, and stored as individual 16-bit uncompressed TIFF files per imaged site and per channel.

## Computational image analysis

For the primary screen 1.4x10^6^ images (including separate images for both nuclear and virus channel) were analyzed, amounting to 1.2TB of image data. The images were analyzed with open-source image analysis software [[2](#_ENREF_2)] (<http://www.cellprofiler.org>), combined with previously published software for supervised classification of cellular phenotypes [[3](#_ENREF_3)], and image analysis methods described previously [[1](#_ENREF_1),[4](#_ENREF_4)]. All computational image analysis was run on the high-performance computing cluster Brutus, and data storage on the central IT services, both from the ETH Zurich. The analysis resulted in 500GB of MATLAB analysis result files, describing ~200 measurements for each of 442,744,654 cells.

### CellProfiler Image analysis

The CellProfiler [[2](#_ENREF_2)] (<http://www.cellprofiler.org>) image analysis pipeline was as follows: first, nuclei were identified based on the DAPI stain. Next, cell boundaries were estimated using nuclear expansion. Standard CellProfiler texture, intensity, size, and shape features were extracted from nucleus and cell regions, as well as from complete images, for all available channels. In total, around 200 features were measured per individual cell.

### Supervised classification of cellular phenotypes

We applied supervised machine learning using the open source support vector machine (SVM) learning tool CellClassifier [[3](#_ENREF_3)] (<http://www.cellclassifier.ethz.ch>), to identify biologically and technically relevant cellular phenotypes. We classified all cells in each screen into 5 distinct binary phenotype classes (infected/non-infected, interphase/non-interphase, mitotic/non-mitotic, apoptotic/non-apoptotic, blob/non-blob). Classification was performed as described [[1](#_ENREF_1),[4](#_ENREF_4)]. In total, 442x10^6^ cells were analyzed, of which approximately 15% were discarded by the SVM based selection (cell segmentation errors, out-of-focus image regions, and experimental contaminants). After SVM cleanup, the median correlation coefficient for infection over replicate plates was 0.79, and for the total number of cells per well it was 0.69, indicating a high reproducibility of the applied screening pipeline.

### Hit scoring

We first tested, if we should apply a normalization for the influence of population context determined parameter such as local cell density. However, our results [[4](#_ENREF_4)] showed that for this screen in particular no improvement regarding siRNA reproducibility was observed after population context correction, and we therefore decided to work with data that was not corrected for the influence of population context. Genes were scored according their changed level of infection after several steps of data normalization. Fold-change of infection was calculated per well as the log2 transform of the measured infection divided over the median level of infection for all wells of that plate. These fold-changes of infection were next corrected for column and row-wise plate effects, and plate-wise z-score normalized (subtract plate mean, then divide by plate SD). The median of the triplicate z-scores was determined for each individual siRNA. As three siRNAs per gene were used, we next calculated the median of the three siRNAs scores corresponding to each gene. The genes were then ranked from lowest to highest based on these median-of-median scores, and genes


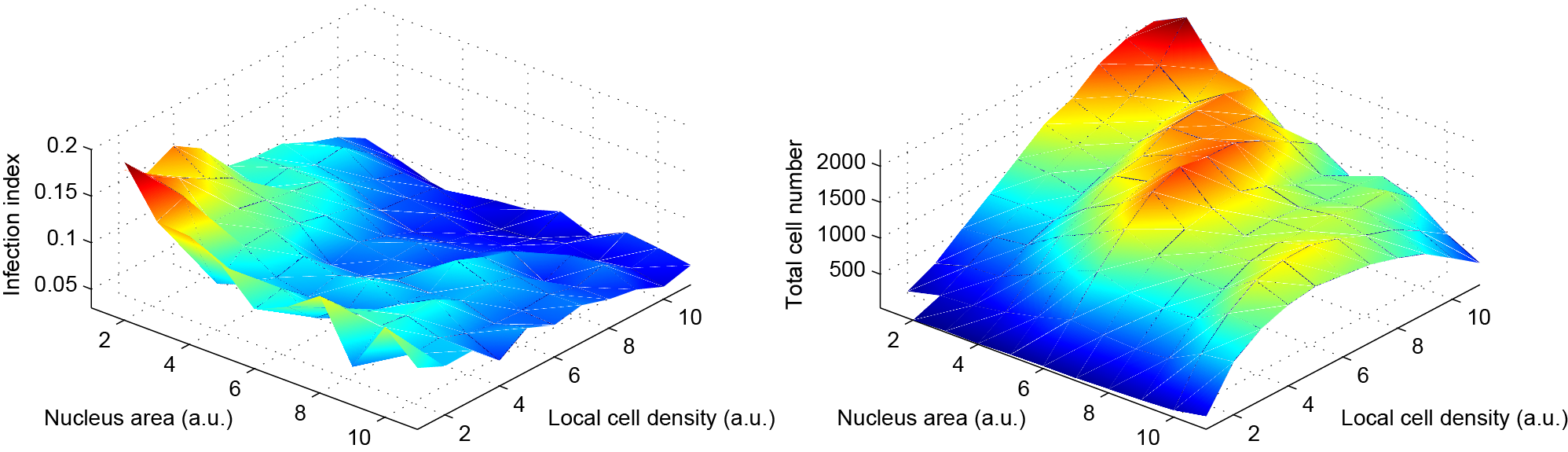


Typical population context dependent HPV infection observed

for a multi-well plate in the primary screen.

## Secondary Screen

The secondary screen was performed in three independent experiments of duplicates with three independent siRNAs from a different vendor (Ambion). The siRNAs were randomly spotted on 96-well plates for reverse transfection as above. Each of the twelve plates of the secondary screen contained four siRNA transfection controls (cytotoxic siRNA), four scrambled controls (scrambled siRNA), four anti-reporter controls (RNAi against GFP) and eight no-virus controls (wells to which no virus was added). Reverse transfection of siRNA was performed by seeding the cells into a well containing 20µl of transfection mix. The transfection mix for one well contained 0.1µl Lipofectamine RNAimax (Invitrogen), 14.9µl Optimem and 5µl of 1µM siRNA (diluted in RNase free ddH2O). In each well, 3000 cells were seeded in 50 µl/well of complete medium. Cells were then left at 37^o^C for 60h prior to infection.

Infection and staining were carried out as in the primary screen. Imaging was performed on a Pathway 855 automated microscopy station (Becton Dickinson) using a 10x objective (Olympus) employing a laser-based autofocus every image. Cell numbers and raw infection indices for each well were determined using a MATLAB-based infection scoring procedure (The Mathworks) described previously [[5](#_ENREF_5)]. The raw infection indices were normalized to scrambled siRNA transfected control cells to result in relative infection percentages. Otherwise hit scoring was carried out as for the primary screen. Validated hits were defined as positive for both primary and secondary screen.

### Integration of host factors with functional gene interactions and annotations

Functional annotation clustering of DAVID was combined with the functional interactions described in the STRING database ([www.string-db.org](http://www.string-db.org)), as described in [[6](#_ENREF_6)].

**Cell cycle analysis**

HeLa cells were treated with cell cycle inhibitors as above for 24h. Cells were trypsinized, pelleted, and fixed overnight in cold 70% ethanol. Cells were washed thrice with PBS, treated with RNase cocktail (Ambion), incubated with 50µg/ml propidium iodide (Sigma) for 15min, and the DNA content was determined after flow cytometry analysis using FlowJo (v9.2).

**HPV16 internalization assay**

2.5x10^5^ HeLa cells were treated for 16h with aphidicolin, after which AF488-HPV16 PsV (100 vge/cell) were allowed to bind for 1 h, the inoculum was removed, and internalization was analyzed at 6h p.i. in the presence or absence of aphidicolin. To determine the total amount of cell associated virus, cells were detached with non-enzymatic cell dissociation solution (Sigma), whereas for internalized virus, cells were treated with 0.25% Trypsin/EDTA (Gibco) supplemented with 50 µg/ml Proteinase K (Roche) effectively removing surface-bound virus. Cells were fixed with 4% PFA and cell-associated fluorescence was measured by flow cytometry.

**IBB-GFP nuclear import assay**

10^5^ HeLa IBB-GFP cells were seeded on glass cover slips, treated with 15µM aphidicolin and 10µg/ml cycloheximide to reduce the IBB-GFP signal for 18h, after which cycloheximide or both inhibitors were washed out. Cells were fixed in 4% PFA 10h after washout, and nuclei were stained with Hoechst. Microscopic images were acquired with a Hamamatsu EM-CCD digital camera connected to an Olympus IX70 inverted microscope using a 20x objective and monochromator (Till Photonics) excitation.

**Supplemental references**

1. Snijder B, Sacher R, Ramo P, Damm EM, Liberali P, et al. (2009) Population context determines cell-to-cell variability in endocytosis and virus infection. Nature 461: 520-523.

2. Carpenter AE, Jones TR, Lamprecht MR, Clarke C, Kang IH, et al. (2006) CellProfiler: image analysis software for identifying and quantifying cell phenotypes. Genome Biol 7: R100.

3. Ramo P, Sacher R, Snijder B, Begemann B, Pelkmans L (2009) CellClassifier: supervised learning of cellular phenotypes. Bioinformatics 25: 3028-3030.

4. Snijder B, Sacher R, Ramo P, Liberali P, Mench K, et al. (2012) Single-cell analysis of population context advances RNAi screening at multiple levels. Mol Syst Biol 8: 579.

5. Engel S, Heger T, Mancini R, Herzog F, Kartenbeck J, et al. (2011) Role of endosomes in simian virus 40 entry and infection. J Virol 85: 4198-4211.

6. Mercer J, Snijder B, Sacher R, Burkard C, Bleck CK, et al. (2012) RNAi screening reveals proteasome- and Cullin3-dependent stages in vaccinia virus infection. Cell Rep 2: 1036-1047.
